# Single‐cell and spatial analyses reveal the association between gene expression of glutamine synthetase with the immunosuppressive phenotype of APOE+CTSZ+TAM in cancers

**DOI:** 10.1002/1878-0261.13373

**Published:** 2023-01-21

**Authors:** Jinfen Wei, Wenqi Yu, Juanzhi Chen, Guanda Huang, Lingjie Zhang, Zixi Chen, Meiling Hu, Xiaocheng Gong, Hongli Du

**Affiliations:** ^1^ School of Biology and Biological Engineering South China University of Technology Guangzhou China; ^2^ Department of Pathology, School of Basic Medical Sciences Southern Medical University Guangzhou China; ^3^ Department of Pathology Nanfang Hospital, Southern Medical University Guangzhou China

**Keywords:** colorectal cancer, GLUL, immunosuppressive microenvironment, metabolism, single‐cell and spatial RNA‐seq, tumor‐associated macrophages

## Abstract

An immunosuppressive state is regulated by various factors in the tumor microenvironment (TME), including, but not limited to, metabolic plasticity of immunosuppressive cells and cytokines secreted by these cells. We used single‐cell RNA‐sequencing (scRNA‐seq) data and applied single‐cell flux estimation analysis to characterize the link between metabolism and cellular function within the hypoxic TME of colorectal (CRC) and lung cancer. In terms of metabolic heterogeneity, we found myeloid cells potentially inclined to accumulate glutamine but tumor cells inclined to accumulate glutamate. In particular, we uncovered a tumor‐associated macrophage (TAM) subpopulation, APOE+CTSZ+TAM, that was present in high proportions in tumor samples and exhibited immunosuppressive characteristics through upregulating the expression of anti‐inflammatory genes. The proportion of APOE+CTSZ+TAM and regulatory T cells (Treg) were positively correlated across CRC scRNA‐seq samples. APOE+CTSZ+TAM potentially interacted with Treg via CXCL16–CCR6 signals, as seen by ligand–receptor interactions analysis. Notably, glutamate‐to‐glutamine metabolic flux score and glutamine synthetase (*GLUL*) expression were uniquely higher in APOE+CTSZ+TAM, compared with other cell types within the TME. *GLUL* expression in macrophages was positively correlated with anti‐inflammatory score and was higher in high‐grade and invasive tumor samples. Moreover, spatial transcriptome and multiplex immunofluorescence staining of samples showed that APOE+CTSZ+TAM and Treg potentially colocalized in the tissue sections from CRC clinical samples. These results highlight the specific role and metabolic characteristic of the APOE+CTSZ+TAM subpopulation and provide a new perspective for macrophage subcluster‐targeted therapeutic interventions or metabolic checkpoint‐based cancer therapies.

## Graphical abstract

Using single‐cell flux estimation analysis, spatial transcriptome, and multiple immunofluorescence analysis, we uncovered a cell subpopulation, APOE+CTSZ+TAM, that exhibited immunosuppressive characteristics through upregulating the expression of anti‐inflammatory genes and communicating with Treg cells via CXCL16–CCR6 signals. Glutamate‐to‐glutamine metabolic flux score and glutamine synthetase expression were uniquely higher in APOE+CTSZ+TAM, indicating that regulating metabolism may help reprogram APOE+CTSZ+TAM to an antitumor phenotype.

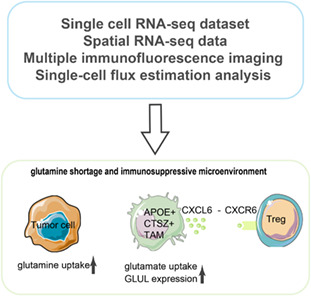

**Abbreviations**
CRC: colorectal cancer
DEGs: differentially expressed genes
EMT: epithelial–mesenchymal transition
FFPE: formalin fixed and paraffin embedded
GLS: glutaminase
GLUD1: glutamate dehydrogenase 1
GLUL: glutamate–ammonia ligase
LC: lung cancer
MDSC: myeloid‐derived suppressor cell
mIF: multiplex immunofluorescence
MMRd: mismatch repair deficient
MMRp: mismatch repair proficient
OGDH: oxoglutarate dehydrogenase
scRNA‐seq: single‐cell RNA sequencing
SLC3A1: solute carrier family 3 member 1
ssGSEA: single sample gene set enrichment analysis
ST: spatial transcriptome
SUCLG1: succinyl‐CoA synthetase subunit alpha
TAM: tumor‐associated macrophage
TME: tumor microenvironment
Treg: regulatory T cells
UMAP: uniform manifold approximation and projection
UMI: unique molecular identifiers

## 1 Introduction

Forming the immunosuppressive microenvironment is key to tumor immune escape and malignant tumor progression, which is triggered by various factors, including hypoxia and the metabolic composition in the tumor microenvironment (TME). For example, methionine depletion in the TME causes T cell persistently expressing inhibitory receptors and further promotes tumor progression in hepatic carcinoma [1] and colon cancer [2]. Besides, hypoxia, a common characteristic in solid tumors and associated with poor prognosis in several cancer types [3, 4], negatively affects antitumor immune responses by reducing activity of effector cells and by increasing the recruitment of immunosuppressive cells and supporting their activity [5]. Thus, it is critical to understand the metabolic signals on exacerbating the immunosuppressive microenvironment and destroying the antitumor immune response, as the activation and differentiation of immune cells occur in metabolically reprogrammed TME in cancers [6].

Additionally, myeloid‐derived suppressor cell (MDSC) [7], tumor‐associated macrophage (TAM) [8], and regulatory T cell (Treg) [9] within the TME harbor a highly immunosuppressive phenotype, inducing repressed function of effector T cells and influencing antitumor immunity. Importantly, these immunosuppressive cells are metabolically flexible, can use alternative metabolites in the TME to maintain their suppressive identity and help cancer cells evade the immune system. For example, TAM could adopt phenotypes to immunosuppressive and maintain cell vitality by using lactic acid released by tumor cells [10], thereby directly inhibiting the cytotoxicity of T cells. TAM could destroy antitumor immunity through releasing arginase‐1 and depletion of l‐arginine which is essential for the re‐expression of the T‐cell receptor after antigen engagement on T cells [11]. The exploitation of targeted therapies against TAM and the strategies including depletion and reprogramming macrophages have been implemented in preclinical and clinical trials [12]. Despite these advances, the strategy needs further investigation for its limitations. For example, the general depletion of macrophages exerted by CSF1R inhibitors is not TAM specific and thus has substantial toxicity over time [13]. Identification of metabolic checkpoints of macrophages' function might represent a promising strategy to induce selective reprogramming of abundant protumoral macrophages toward an antitumoral phenotype [14]. Although different functional macrophages are known to localize in different metabolic environments of the tumor, how the specific metabolic features regulating TAM subgroups behavior *in vivo* of clinical samples and affecting their corresponding impact on disease outcome has not been well studied.

In the current study, as single‐cell RNA‐sequencing (scRNA‐seq) data has been instrumental in understanding heterogeneity and identifying novel metabolic regulators of cells [15], we took advantage of scRNA‐seq to reveal the characteristics and function of individual cell and depict the metabolic profile in the hypoxia landscape across cell types. We applied scRNA‐seq data from colon cancer (CRC) and lung cancer (LC) which were the cancer types to harbor hypoxia characteristics [3, 16]. To analyze these data, we utilized a novel computational method, single‐cell flux estimation analysis (scFEA), to assess metabolic profile from scRNA‐seq data. The results showed that APOE+CTSZ+TAM, with significant anti‐inflammatory phenotype and specifically expressing *GLUL*, was associated with malignant progression in colon cancer patients. This study highlights that GLUL may be a macrophagic metabolic checkpoint as a promising alternative to tackle immunosuppressive and tumor progression.

## 2 Materials and methods

### 2.1 Data collection and human specimens

The single‐cell gene expression matrices in the present study were retrieved from the following databases: CRC Single Cell Portal (https://singlecell.broadinstitute.org/single_cell/study/SCP1162) [17], LC1 (GSE131907) [18], and LC2 (http://blueprint.lambrechtslab.org) [19]. CRC single‐cell data were divided into two groups according to the samples from mismatch repair‐deficient (MMRd) and mismatch repair‐proficient (MMRp) patients, named CRC‐MMRd and CRC‐MMRp respectively. Spatial transcriptome (ST) data were available from CNGB Nucleotide Sequence Archive (CNSA: http://db.cngb.org, accession number CNP0002432).

Nine CRC tumor specimens from Nanfang Hospital, Guangzhou, China, were included in this study for multiplex immunofluorescence (mIF). The clinical characteristics of patients for mIF are shown in Table S1. This study was approved by the Ethics Committee of Nanfang Hospital of Southern Medical University (Approval No. NFEC‐2021‐110) and complied with the Declaration of Helsinki. The experiments were undertaken with the understanding and written consent of each subject.

### 2.2 Single‐cell RNA‐seq data processing

The raw gene expression matrices were processed using seurat (v4.1.0) r package. In the quality control steps, the following genes or cells were filtered out: (a) genes expressed by < 50 cells; (b) cells < 800 or cells > 6000 expressed genes; and (c) cells > 20% of mitochondrial genes.

We constructed principal components (PCs) using highly variable genes, then selected the first 30 PCs for graph‐based clustering with functions FindNeighbors and FindClusters in seurat. We performed harmony algorithm in harmony r package [20] to remove batch correction before subclustering analysis of epithelial cells. For visualization of clustering analysis, we performed uniform manifold approximation and projection (UMAP) using RunUMAP function in seurat.

### 2.3 Cell‐type annotation and differential expression analysis

We discriminated differentially expressed genes (DEGs) based on Wilcoxon rank‐sum test using the ‘FindAllMarkers’ function in the seurat by comparing the difference in each cluster to the union of the rest of the clusters. Genes with *P* value < 0.05 were considered as DEGs based on Bonferroni correction. We used heatmap to visualize DEGs of subcluster based on gene expression after the log‐transformed and scaling.

The clusters and subclusters were annotated based on the top‐ranking DEGs among the canonical marker genes known from previous studies. Detailed information on cluster including major cluster, subcluster, corresponding marker genes, and cell numbers were addressed in Tables S2 and S3.

### 2.4 Definition of gene signature scores involved in cell‐specific function and cancer hallmarks

To compare the transcriptional signatures between cells, we used the gene sets from MsigDB (http://www.gsea‐msigdb.org/gsea/msigdb/) to define the epithelial–mesenchymal transition (EMT) by calculating single‐sample gene set enrichment analysis (ssGSEA) score. The gene sets of metabolism pathway were obtained from a previous study [21]. The gene signatures (CD8 T‐cell activation, cytotoxicity, exhaustion activity, M1/M2 macrophages, proinflammatory, and anti‐inflammatory) were obtained from previous research to distinguish the features of each cluster in T/NK cells and myeloid cells, respectively [22, 23]. Hypoxia score was calculated by ssGSEA method using gene signatures referring to our previous study [16]. For each dataset, the cells were divided into hypoxia‐high (top 50%) and hypoxia‐low (bottom 50%) groups according to the hypoxia score. As for the gene signature of specific cell types identified by the current study, we also adopted ssGSEA method to calculate the scores of specific cell type. The signature scores were calculated across all cells in each cancer dataset separately. All gene signatures were listed in Table S4.

### 2.5 Cell–cell interaction analysis

In order to reveal the molecular mechanism of crosstalk between cells in the TME, Cellphone DB [24] was used to infer the ligand–receptor interaction between cell clusters. The ligands and receptors pairs are shown in a bubble chart for showing the average genes' expression in pairs and the *P* value.

### 2.6 Survival analysis

The samples were grouped into high and low groups according to the specific gene expression. For *APOE* and *CTSZ* as well as *GLUL* gene expression, we performed survival analysis using the top and bottom 50% expression as high and low groups in the online website, using a standard professing pipeline (http://gepia2.cancer‐pku.cn/) [25].

### 2.7 Detecting metabolic modules and metabolomic changes in each cell type

We applied scFEA method [26] to infer the cell‐wise metabolic flux from scRNA‐seq data and to identify context‐ and cell types‐specific metabolic diversities. Tissue‐level metabolic stress was computed as the total imbalance throughout the cells. A neural network in scFEA includes three hidden layers and each layer with eight hidden nodes. Hyperbolic tangent served as activation function and the neural network defines the loss function considered from four parts, including flux balance loss, non‐negative loss, inconsistency with gene expression, and flux scale.

In the data processing step, the neural network returned a row with the module score in each cell. We accumulated the sum of consumption score and subtracted the sum of production score of each module in a cell, thus obtaining the metabolism score of each compound corresponding to the cell. We used the average score among all cells in this cluster for the metabolic score of specific cell cluster.

### 2.8 Processing and analysis of spatial transcriptome data

The ST data used in this study were obtained from a previous study, which was sequenced in the Stereo‐seq platform [27]. We summarized the unique molecular identifiers (UMI) in each of the bin100‐defined (50 μm × 50 μm) spots, and then analyzed and visualized the results. For determining colocalization of Macro_APOE/CTSZ and Treg, the signature score was calculated by ssGSEA method across all spots in the tissue section. The correlation between signature score of APOE+CTSZ+TAM and Treg was calculated using Spearman correlation method. Scanpy [28] was used for spatial visualization of cell types and gene expression.

### 2.9 Multiplex immunofluorescence imaging and image analysis

Formalin‐fixed and paraffin‐embedded (FFPE) tissue blocks were chosen from nine CRC patients in Nanfang Hospital for subsequent analysis. Immunostaining was processed with Opal™ 7‐Color IHC Kits (Akoya Biosciences, Marlborough, MA, USA; NEL821001KT), according to the manufacturer's instructions using antibodies (anti‐human CD68 (Proteintech, Chicago, IL, USA; Cat# 66231‐2‐Ig, RRID: AB_2881622); anti‐human APOE (Proteintech; Cat# 66830‐1‐Ig, RRID: AB_2882173); anti‐human CTSZ (Affinity Biosciences, Melbourne, VIC, Australia; Cat# DF14386, RRID:AB_2923130); anti‐human GLUL (Affinity Biosciences; Cat# DF7607, RRID: AB_2841098), and anti‐human FOXP3 (Affinity Biosciences; Cat# AF6544, RRID: AB_2847268)). Images were obtained and analyzed with the Zeiss LSM 880 confocal laser‐scanning microscope (LSM 880; Carl Zeiss, Oberkochen, Germany) and ZEN image analysis software (zen; Carl Zeiss). imagej was used to perform the fluorescence quantitative colocalization analysis [29].

### 2.10 Statistical analysis

All statistical analyses and graphical representations of data were performed in the r and python computational environment. The correlation analysis including gene expression, gene signature score, and cell proportion between two groups used in this study was based on Spearman correlation. For the cell subtype abundance correlation matrix, we defined the number ratio of cell subtype to the belonging major cell type as the relative abundance of each cell subtype, then computed the Spearman correlation coefficient between the relative abundance of all cell subtypes across CRC patients and considered *r* > 0.3 and FDR < 0.05 as significant correlation. For the difference analysis between groups, we used the Kruskal–Wallis test to compare multiple groups, and a two‐sided Wilcoxon rank‐sum test to perform pairwise comparisons. For the pseudo‐bulk analyses [30], we grouped cells in one patient as a pseudo‐bulk, and then compared the variance in the value of gene expression, gene signature score, metabolism model score, and cell proportion between pseudo‐bulks. In all cases, statistical significance was defined as an adjusted *P* value < 0.05. Details of all statistical tests used can be found in the corresponding figure legends.

## 3 Results

### 3.1 Cell types and subtypes within hypoxia TME across CRC and LC

**Fig. 1:**
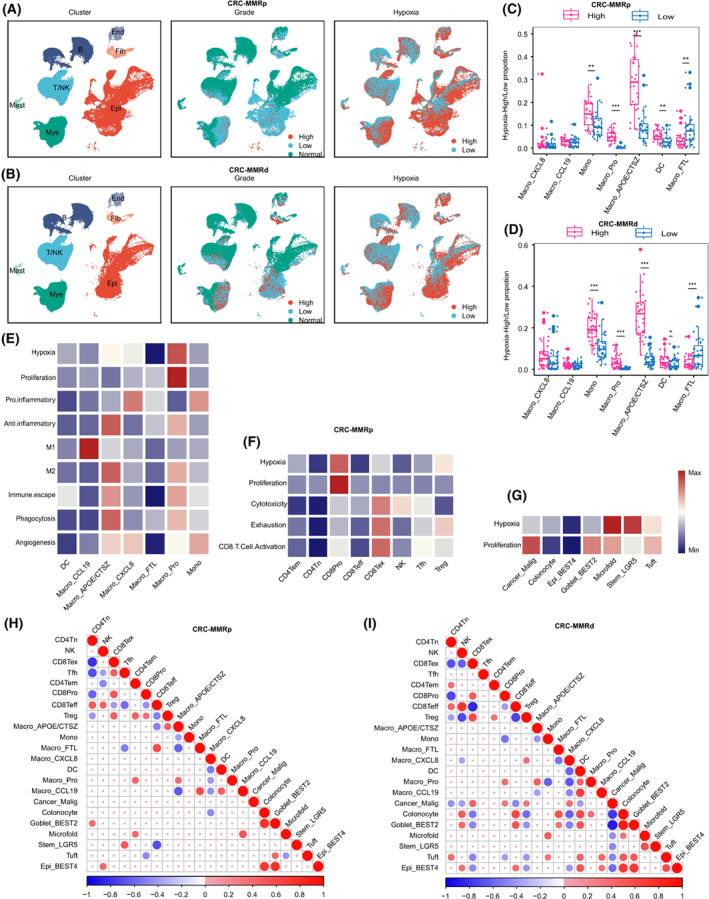
Single‐cell atlas of human CRC tissues. (A) UMAP plots of cells from normal and tumor tissue of 28 CRC‐MMRp patients, showing seven clusters indicating cell type, three clusters indicating tumor staging information, and two clusters indicating hypoxia‐high and ‐low group cells. Each cluster was shown in different colors. (B) The same as shown in A but in 34 CRC‐MMRd patients. (C, D) Cell proportion in hypoxia‐high and ‐low groups of each cell type in CRC‐MMRp (C) and CRC‐MMRd (D). **P < 0.01, ***P < 0.001, paired two‐sided Wilcoxon test. The mean cell proportion is marked in the bar graph and vertical lines indicate the maximum and minimum values of the cell proportion. (E–G) Heatmap showing different expression patterns of cell function‐associated gene signatures among myeloid cell subsets (E), in T/NK cells (F), and in epithelial cells (G). (H, I) Correlation map showing the correlation between cellular proportion with positive (Spearman correlation; correlation coefficient *r* > 0.3 and FDR < 0.05, in red), negative (*r* < −0.3 and FDR < 0.05, in blue), or nonsignificant (blank) correlation for the infiltration of pairwise 22 cell types in 28 independent CRC‐MMRp samples (H) and 24 CRC‐MMRd samples (I). MMRd, mismatch repair deficient; MMRp, mismatch repair proficient; Mono, monocyte; DC, dendritic cell; NK, natural killer; Tfh, follicular helper T cell; Mye, myeloid cells; Epi, epithelial cells.

Using gene expression of canonical marker, the cell types and subtypes were identified across CRC‐MMRd, CRC‐MMRp, LC1, and LC2 datasets, respectively (Fig. S1a–c, Tables S2 and S3). All cells were divided into hypoxia‐high or hypoxia‐low group in each dataset by using hypoxia gene signature score to characterize hypoxia level. Notably, T/NK cells were mainly in hypoxia‐low group while myeloid and epithelial cells were in hypoxia‐high group (Fig. 1A,B; Fig. S1d–f). After comparing the hypoxia level between subtypes, we found that Mono and Macro_APOE/CTSZ subclusters were highly enriched in hypoxia‐high group, while Macro_FTL subcluster was mainly in hypoxia‐low group in both CRC‐MMRp and CRC‐MMRd datasets (Fig. 1C,D). As hypoxia‐inducible factor‐1 (HIF‐1A) was one of the indicators of hypoxia, we further calculated the gene expression between *HIF1A*, *APOE*, and *CTSZ*. The results showed that there was a significant correlation between them in both CRC and LC, except no correlation between *APOE* and *HIF1A* in CRC‐MMRd samples (Fig. S1g).

To obtain an in‐depth understanding of the cellular function of each cell subtype, we performed ssGSEA analysis using gene signatures and compared the similarities and differences between subclusters. In the myeloid cells, Macro_APOE/CTSZ was observed to have higher anti‐inflammatory and M2‐like scores in CRC‐MMRp (Fig. 1E). In the T/NK cells, hypoxia and proliferation scores were higher in CD8Pro subcluster, while exhaustion score was higher in CD8Tex subcluster (Fig. 1F). In the epithelial cells, Cancer_Malig subcluster with a higher proliferation score was observed in CRC‐MMRp (Fig. 1G). Similar results were observed in CRC‐MMRd, LC1, and LC2 datasets (Fig. S2a–c).

To explore the potential synergistic effects of different functional cells in promoting cancer, we next aimed to calculate the infiltration proportion correlation between these cells. Due to the limited number of LC samples, only CRC was selected in the current analysis. In general, the proportion of cell subclusters was correlated between cells in the same major cell types. For example, there was a positive correlation between epithelial cell subclusters in both CRC‐MMRp (Fig. 1H) and CRC‐MMRd (Fig. 1I). Notably, Macro_APOE/CTSZ was observed to show a positive correlation with Treg, but a negative correlation with CD8Teff subcluster in CRC‐MMRp (Fig. 1H).

### 3.2 Metabolic heterogeneity across cell subclusters and association between metabolic characteristics and cellular function

**Fig. 2:**
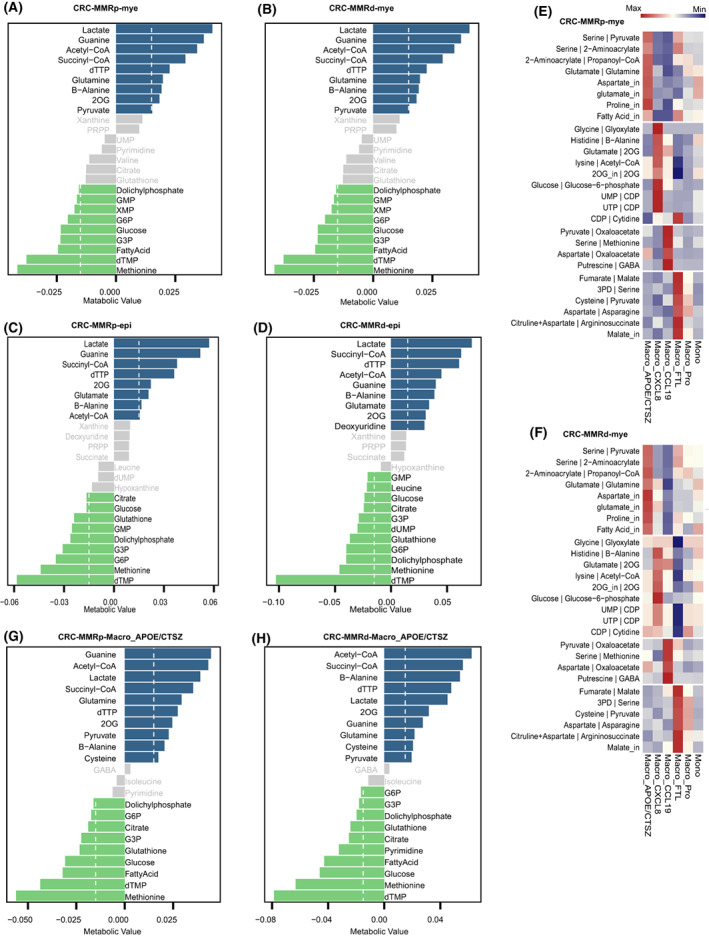
The metabolic characteristics across single cells. (A, B) Top accumulated and depleted metabolites predicted in the myeloid cells in CRC‐MMRp samples (A) and CRC‐MMRd samples (B). The *x*‐axis is metabolism stress level, where a positive value represents accumulation and a negative value represents depletion. (C, D) Top accumulated and depleted metabolites predicted in the epithelial cells in CRC‐MMRp samples (C) and CRC‐MMRd samples (D). (E, F) Distribution of predicted cell‐wise flux of metabolism in the subtypes of myeloid cells in CRC‐MMRp samples (E) and CRC‐MMRd samples (F). (G, H) Top accumulated and depleted metabolites predicted in the APOE+CTSZ+TAM in CRC‐MMRp samples (G) and CRC‐MMRd samples (H). The dashed line in A–D, G, and H shows the value of accumulated or depleted metabolites equaling 0.015. The values less than this value are gray bars.

Owing to the lack of matched metabolomics information, we focused on demonstrating the capability of scFEA in inferring metabolic flux, metabolic stress, and metabolic modules between cell subclusters. We performed pseudo‐bulk analyses and differential analysis to reveal the differences between cell subclusters and to reflect the variability between patients. The results showed that there were notable differences between cell types, for example, epithelial cells were revealed to have the highest level in most metabolic reactions compared to other cell types in CRC, followed by myeloid cells (Fig. S3a). We then mainly focused on the epithelial cells and myeloid cells and compared the difference among subclusters belong to these two cell types. The top accumulated metabolites were lactate and top depleted metabolites were methionine and dTMP in myeloid cells (Fig. 2A,B) and epithelial cells (Fig. 2C,D). Notably, glutamine and glutamate were one of the top accumulated metabolites observed in myeloid cells and epithelial cells, respectively, which was distinct between these two cell types (Fig. 2A–D). The same results were also observed in LC1 and LC2 samples (Fig. S3b,c).

In the subtypes, Macro_APOE/CTSZ was found to have a higher value in glutamate‐to‐glutamine and glutamate input transport metabolic flux (Fig. 2E,F), which was then confirmed by the observation of accumulated glutamine in Macro_APOE/CTSZ from CRC (Fig. 2G,H) and LC (Fig. S3d). The value of pyruvate‐to‐oxaloacetate and serine‐to‐methionine metabolic flux was higher in Macro_CCL19 subcluster (Fig. S3e). The value of glycolysis‐related metabolic flux was higher in Cancer_Pro subcluster (Fig. S3f). Pseudo‐bulk analyses by grouping cells from distinct patients also showed significant differences between subclusters, in different metabolic fluxes (Fig. S4a,b). These results reveal that diverse cell populations might preferentially acquire distinct metabolites from a common pool of metabolites available in the TME.

Since the metabolic characteristics differed between cell subtypes with different functions, we further analyzed the relationship between metabolic features and cellular function across cell types. In macrophages, ssGSEA score of amino acid metabolic pathway was positively correlated with score of anti‐inflammatory, M2‐like polarization and immune escape score signatures (Fig. S5a), however, they were negatively correlated with pro‐inflammatory signature score (Fig. S5b). Notably, glutamate‐to‐glutamine flux score was positively correlated with anti‐inflammatory score across LC and CRC datasets (Fig. S5b). As the function of epithelial cells, we observed that score of most metabolism pathways was significantly negatively correlated with EMT score (Fig. S5c). In the T cells, both glycolysis and oxidative phosphorylation score were positively correlated with CD8T cell activation score (Fig. S5d,e). Overall, these data suggest that metabolic heterogeneity between cell types and the relevance between particular metabolism with different cellular functions.

### 3.3 APOE+CTSZ+TAM with an anti‐inflammatory phenotype and associated with high‐grade tumors

**Fig. 3:**
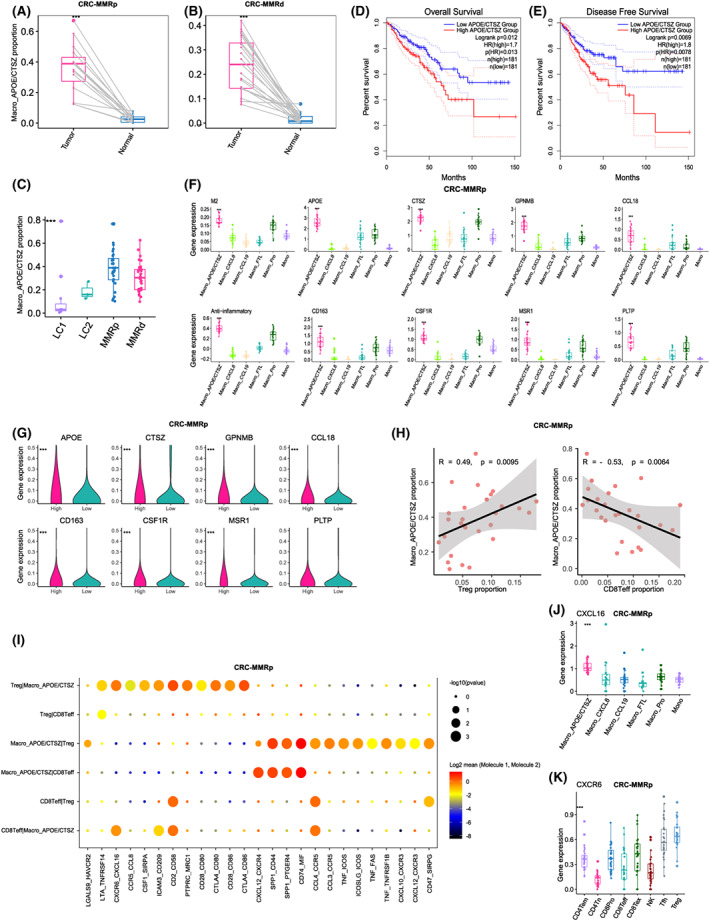
The immunosuppressive function of APOE+CTSZ+TAM. (A, B) Comparison of APOE+CTSZ+TAM percentages in paired normal (*n* = 12) and tumor (*n* = 12) tissue of CRC‐MMRp (A) and CRC‐MMRd samples (B). ***P < 0.001, paired two‐sided Wilcoxon test. (C) Boxplot showing the proportion of APOE+CTSZ+TAM divided by the total macrophage number across CRC and LC samples. The mean cell proportion is marked in the bar graph and vertical lines indicate the maximum and minimum value of the cell proportion. (D) The Kaplan–Meier curve shows overall survival of COADREAD patients with different APOE+CTSZ+TAM infiltration. (E) The Kaplan–Meier curve shows disease‐free survival of COADREAD patients with different APOE+CTSZ+TAM infiltration. (F) Boxplot showing the different expressions of marker genes of APOE+CTSZ+TAM and M2 as well as anti‐inflammatory score among myeloid cells in CRC‐MMRp samples. ***P < 0.001, Kruskal–Wallis test. (G) Violin plots showing the different expressions of marker genes of APOE+CTSZ+TAM between low‐ and high‐grade samples in CRC‐MMRp samples. ***P < 0.001, paired two‐sided Wilcoxon test. (H) Scatterplot showing the Spearman correlation of the proportion of APOE+CTSZ+TAM (divided by the total macrophage number) and Treg cells or CD8+ Teff cells (divided by the total T/NK cell number) in tumor tissues of CRC‐MMRp samples. (I) Bubble chart showing the top predicated ligands expression in APOE+CTSZ+TAM that modulate Tregs by CellPhoneDB. (J) Boxplot showing the different expressions of *CXCL16* among myeloid cell subclusters in CRC‐MMRp samples. ***P < 0.001, Kruskal–Wallis test. (K) Boxplot showing the different expressions of *CXCR6* among T‐cell subclusters in CRC‐MMRp samples. ***P < 0.001, Kruskal–Wallis test. The mean gene expression is marked in the bar graph and vertical lines indicate the maximum and minimum value of the gene expression.

As Macro_APOE/CTSZ subcluster shows the higher anti‐inflammatory and M2‐like score (Fig. 1E), we next investigated the cellular function of Macro_APOE/CTSZ in‐depth. We found that the proportion of Macro_APOE/CTSZ was significantly higher in tumor than normal tissues (Fig. 3A,B) and accounted for more than 20% of macrophages in most CRC patients (Fig. 3C). We analyzed the expression of *APOE* and *CTSZ* within the bulk transcriptomes of CRC and found that the higher level of jointed *APOE* and *CTSZ* expression was correlated with worse overall survival (Fig. 3D) and disease‐free survival in TCGA‐COADREAD (Fig. 3D,E). Analysis of DEGs revealed that known immunosuppressive markers (such as *APOE*, *CTSZ*, *SEPP1*, *MRC1*, and *CD163*) were highly expressed in Macro_APOE/CTSZ compared with other myeloid subclusters in CRC‐MMRp (Fig. 3F), CRC‐MMRp, LC1, and LC2, respectively (Fig. S6a–c). To assess whether Macro_APOE/CTSZ was associated with histologic grade of the tumor, we found these immunosuppressive markers were significantly upregulated in high‐grade tumors compared with low‐grade in CRC‐MMRp and CRC‐MMRp (Fig. 3G; Fig. S6d). Besides, the expression of these makers also significantly upregulated in myeloid cells derived from tumor compared with normal samples (Fig. S6e,f).

As shown in Fig. 1H, the infiltration of Macro_APOE/CTSZ was positively correlated with Treg but negatively correlated with CD8Teff cells in CRC‐MMRp samples (Fig. 3H). To gain further functional insight, we conducted the receptor–ligand interactions analysis and found there were chemokines and their receptors between Macro_APOE/CTSZ and Treg cells, including *CXCL16–CXCR6*, *CCL4–CCR5*, and *CCL3–CCR5* pairs (Fig. 3I). We further found that *CXCL16* was highly expressed in Macro_APOE/CTSZ (Fig. 3J) and *CXCR6* was expressed in Treg (Fig. 3K), which might indicate that Macro_APOE/CTSZ utilized CXCL16 signals to recruit Treg cells to the tumor site, further exacerbating the immunosuppressive TME. Altogether, these results show that the Macro_APOE/CTSZ abundance associates with high‐grade tumor in patients with CRC by contributing to immunosuppressive TME.

### 3.4 APOE+CTSZ+TAM specifically upregulated *GLUL* expression and glutamate‐to‐glutamine metabolic flux score

**Fig. 4:**
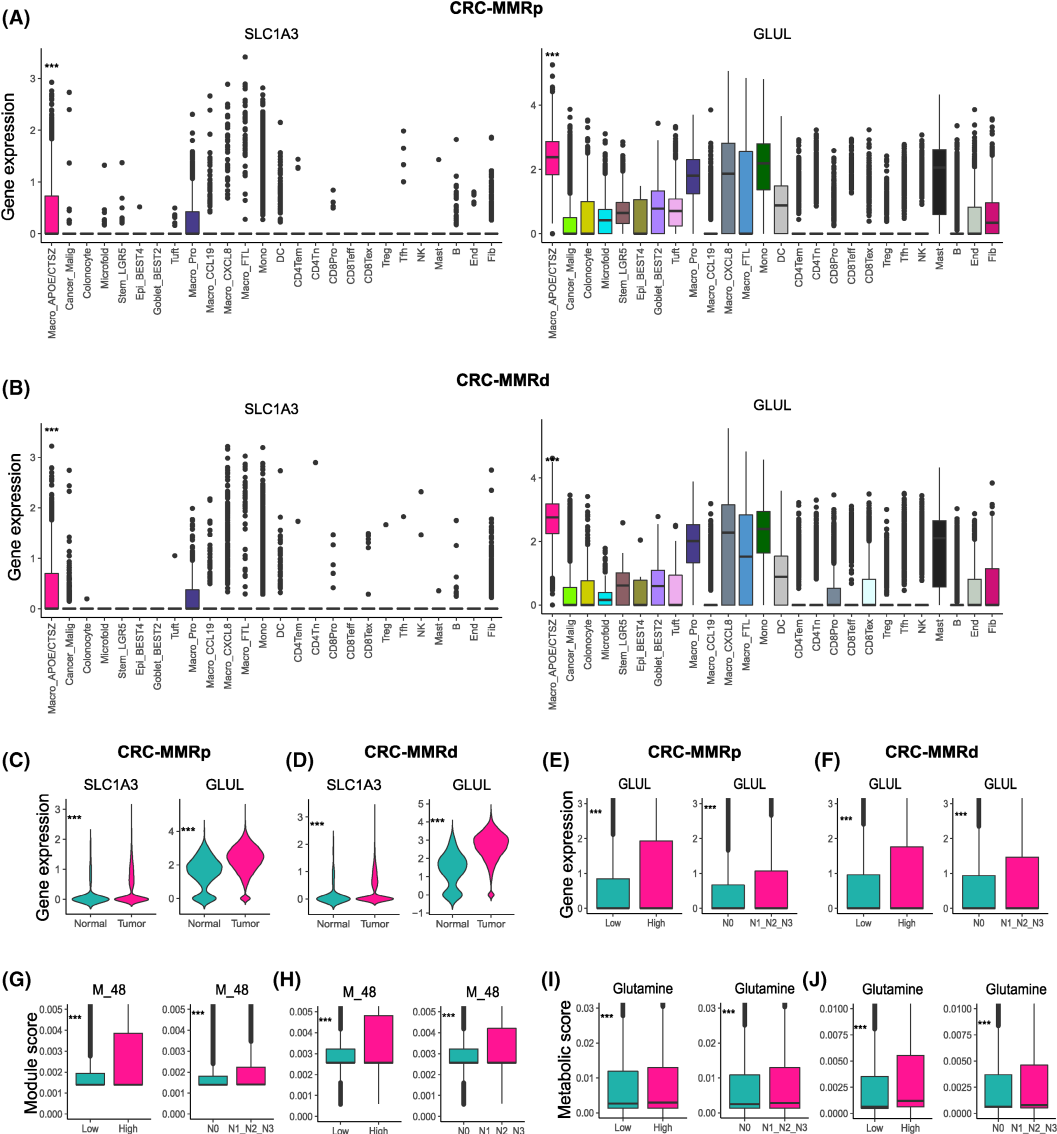
APOE+CTSZ+TAM upregulated GLUL and glutamate‐to‐glutamine metabolic flux. (A, B) *SLC1A3* and *GLUL* expression among cell subtypes in CRC‐MMRp samples (A) and CRC‐MMRd samples (B). ***P < 0.001, Kruskal–Wallis test. The mean gene expression is marked in the bar graph and vertical lines indicate the maximum and minimum values of the gene expression. (C, D) *SLC1A3* and *GLUL* expression in macrophage divided from tumor and normal samples in CRC‐MMRp samples (*n* = 29 in tumor samples, *n* = 29 in normal samples) (C) and CRC‐MMRd samples (*n* = 35 in tumor samples, *n* = 35 in normal samples) (D). (E, F) *GLUL* expression among different histologic grade samples and node status in CRC‐MMRp samples (E) and CRC‐MMRd samples (F). (G, H) M_48 score among different histologic grade samples and node status in CRC‐MMRp samples (G) and CRC‐MMRd samples (H). (I, J) Glutamine accumulation value among different histologic grade samples and node status in CRC‐MMRp samples (I) and CRC‐MMRd samples (J). ***P < 0.001, unpaired one‐sided Wilcoxon test. CRC‐MMRp tumor sample (*n* = 29), CRC‐MMRd sample (*n* = 35), high‐grade CRC‐MMRp tumor sample (*n* = 4), low‐grade CRC‐MMRp tumor sample (*n* = 25), high‐grade CRC‐MMRd tumor sample (*n* = 9), low‐grade CRC‐MMRd tumor sample (*n* = 26), N0 CRC‐MMRp tumor sample (*n* = 12), N1&2&3‐grade CRC‐MMRp tumor sample (*n* = 17), N0 CRC‐MMRd tumor sample (*n* = 23), and N1&2&3‐grade CRC‐MMRd tumor sample (*n* = 12). Mono, monocyte; DC, dendritic cell; NK, natural killer; Tfh, follicular helper T cell; Fib, fibroblast; End, endothelial cell; B, B cell; Mye, myeloid cells; Epi, epithelial cells.

As glutamate‐to‐glutamine metabolic flux (M_48) score was highest in Macro_APOE/CTSZ among all cell subclusters (Fig. 2E,F; Fig. S4), we further explored the link between glutamate‐to‐glutamine metabolic flux and cellular function of Macro_APOE/CTSZ. We evaluated the gene expression of solute carrier family 3 member 1 (*SLC3A1*) and glutamate–ammonia ligase (*GLUL*), which were involved in glutamate transports and glutamine synthase, respectively. The result showed that the gene expression of *SLC3A1* and *GLUL* were highly expressed in Macro_APOE/CTSZ compared with other cell types in CRC and LC, and were upregulated in macrophage derived from tumor than normal tissues in CRC (Fig. 4A–D; Fig. S7b,c). Besides, glutamine accumulation value was observed higher in Macro_APOE/CTSZ subcluster, and was also significantly positively correlated with M_48 score and *GLUL* gene expression in LC and CRC (Fig. S7d,e). Notably, *GLUL* expression (Fig. 4E,F), M_48 score (Fig. 4G,H), and glutamine accumulation value (Fig. 4I,J) were higher in the high‐grade and invasive CRC samples. However, gene expression of *GLUL* was not significantly associated with the overall survival in TCGA CRC and LC cancer patients (Table S5). These results indicate that GLUL expression is remarkably higher in Macro_APOE/CTSZ subcluster and associated with high‐grade tumors.

In order to figure out the underlying causes that Macro_APOE/CTSZ specifically upregulated glutamate transport and glutamate‐to‐glutamine metabolic pathway, we analyzed the glutamate‐related metabolic genes in tumor cells. The results showed that solute carrier family 1 member 5 (*SLC1A5*) and solute carrier family 38 member 1 (*SLC38A1*), encoding enzymes involved in glutamine transports, were specifically upregulated in the tumor cells, while *SLC1A3* and *GLUL* encoding enzymes that were involved in glutamate transports and glutamine synthetase were downregulated in tumor cells compared with Macro_APOE/CTSZ, in LC and CRC (Fig. S7f). Glutaminase (*GLS*), converting glutamine to glutamate, was downregulated in tumor cells compared with Macro_APOE/CTSZ, in CRC samples. However, *GLS* was upregulated in tumor cells derived from cancer compared with epithelial cells derived from normal samples (Fig. S8a,b).

As we observed 2‐OG and succinyl‐CoA were the top metabolites accumulated in the myeloid cells (Fig. 2A–C), we explored whether the accumulation of these metabolites was linked to the overexpression of *GLUL* in myeloid cells. These results showed that *GLS*, glutamate dehydrogenase 1 (*GLUD1*) and oxoglutarate dehydrogenase (*OGDH*), involved in succinyl‐CoA synthesis, were highly expressed in myeloid cells compared with tumor cells in CRC. On the contrary, succinyl‐CoA synthetase subunit alpha (*SUCLG1*), involved in hydrolysis of succinyl‐CoA, was less expressed in myeloid cells compared with tumor cells (Fig. S8c). Besides, these genes were upregulated in myeloid cells derived from tumor compared with myeloid cells derived from normal tissues, in CRC (Fig. S8d). These results indicate that the accumulation of succinyl‐CoA and 2‐oxoglutarate may be partially linked to the overexpression of *GLUL*, but further experiments are needed to verify it in the future work. The above results reveal that Macro_APOE/CTSZ may take measures by upregulating glutamine synthesis pathway in response to glutamine starvation in the TME with glutamine‐addiction tumor cells and to maintain their cellular functions.

### 3.5 Association between glutamate‐to‐glutamine metabolic pathway and anti‐inflammatory function of APOE+CTSZ+TAM

**Fig. 5:**
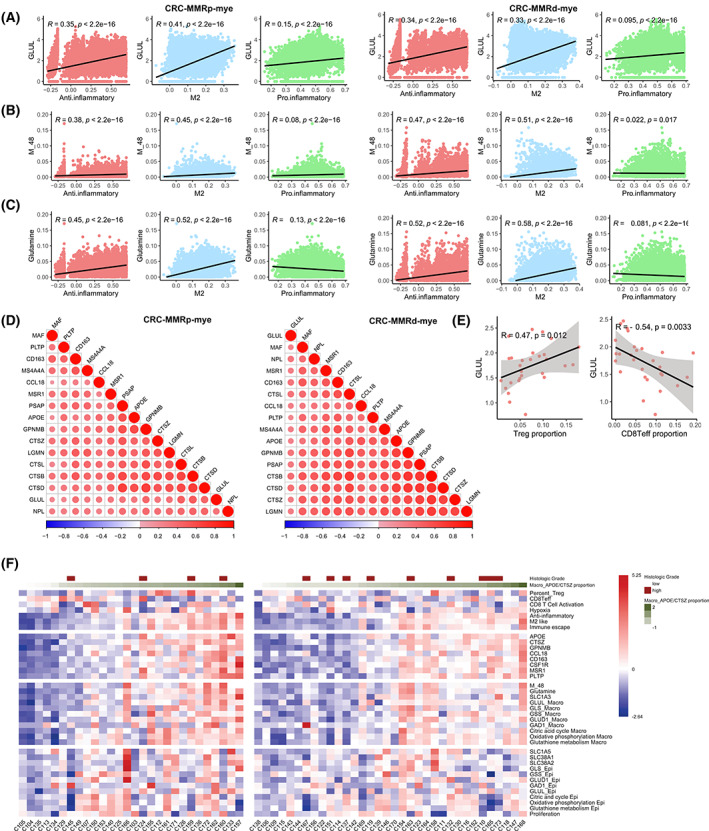
The relevance between metabolism and cell function in macrophages. (A–C) The correlation among GLUL expression (A), M_48 score (B), and glutamine accumulation value (C) with anti‐inflammatory, M2, and proinflammatory score in CRC‐MMRp samples and CRC‐MMRd samples. (D) The expression correlation between *GLUL* with genes in anti‐inflammatory in CRC‐MMRp samples and CRC‐MMRd samples. (E) Scatterplot showing the Spearman correlation of the *GLUL* gene expression and infiltration proportion of Treg or CD8+ T effector cells (divided by the total T/NK cell number) in tumor tissues of CRC‐MMRp samples. CRC‐MMRp tumor sample (*n* = 29) and CRC‐MMRd sample (*n* = 35). (F) Molecular characteristics of different cell types across samples in CRC‐MMRp (left) and CRC‐MMRd patients (right).

Based on the above observations and the immunosuppressive pattern of Macro_APOE/CTSZ, we reasoned that continued glutamine anabolism through GLUL might be responsible for the sustained immunosuppressive phenotype of Macro_APOE/CTSZ. To further explore this hypothesis, we calculated the correlation among the metabolic flux score, anti‐inflammatory score, cell infiltration proportion, as well as the expression of related genes. The results showed that *GLUL* expression, M_48 score, and glutamine accumulated value were positively correlated with anti‐inflammatory score, but negatively or weakly correlated with pro‐inflammatory score across CRC (Fig. 5A–C) and LC (Fig. S9a–c). M_48 score and *GLUL* expression were also highly correlated with anti‐inflammatory‐related genes but weakly or negatively correlated with pro‐inflammatory‐related genes in CRC (Fig. 5D) and LC (Fig. S9d–f). Besides, we found *GLUL* expression was significantly associated with Treg infiltration proportion in CRC‐MMRp samples (Fig. 5E).

In addition, we returned the key molecular characteristics of the above results back to individual samples to inspect their relationship and distribution and found that most of the characteristics in the individuals were consistent with the overall distribution across 28 CRC‐MMRp and 34 CRC‐MMRd patients (Fig. 5F). For example, the extensive association between *GLUL* and anti‐inflammatory score was observed at the individual level along with the abundance change in Macro_APOE/CTSZ. Such findings suggest that glutamine metabolism in the TME may have been shown to promote anti‐inflammatory properties of macrophages and impair the antitumor immunity through critically exacerbated immunosuppression.

### 3.6 Spatial transcriptome and multiple immunohistochemical validation

**Fig. 6:**
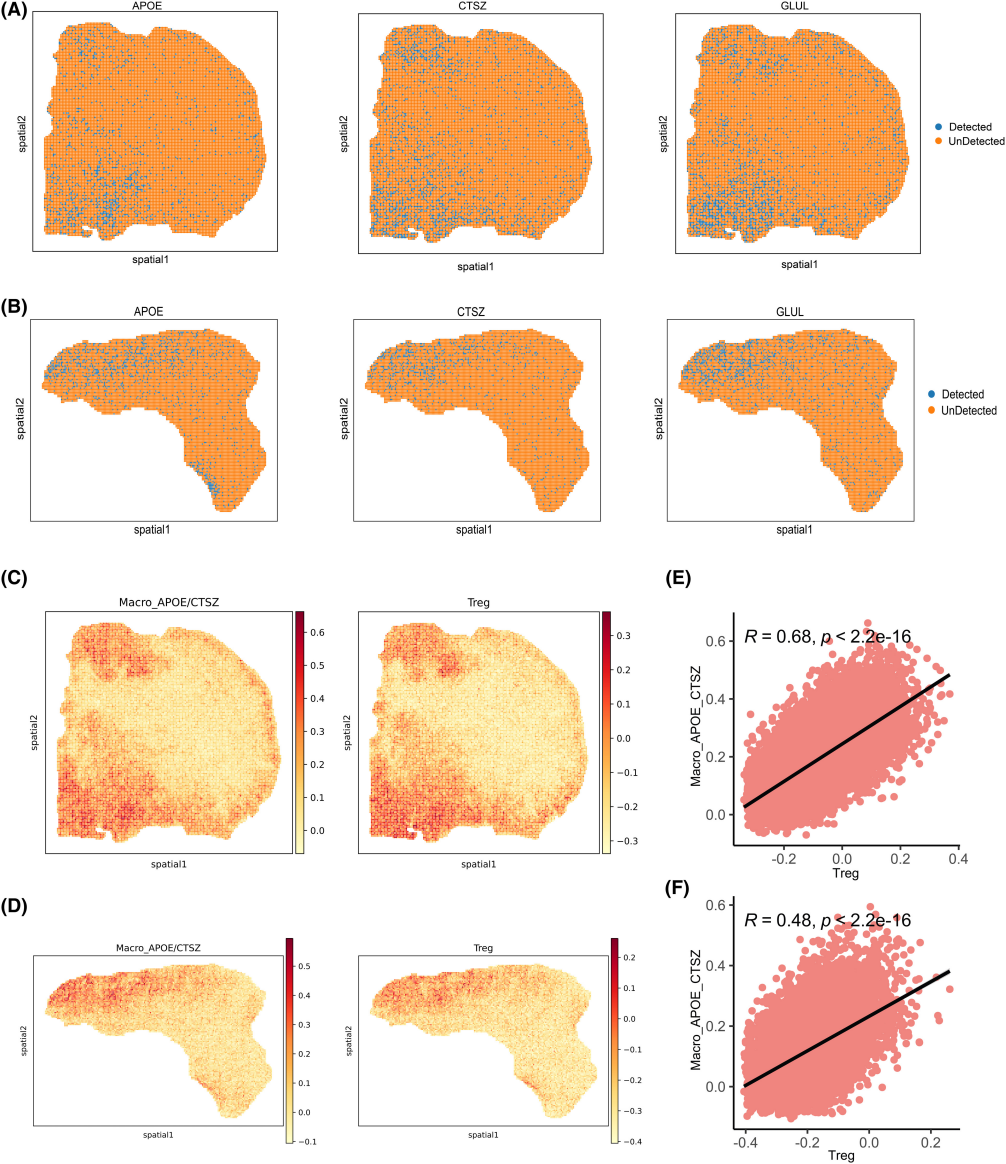
Colocalization of APOE+CTSZ+TAM with cells expressing *GLUL* as well as Treg revealed by spatial transcriptomics. (A, B) Spatial feature plots of gene expression of *APOE*, *CTSZ*, and *GLUL* in patients 19 (A) and 36 (B). (C, D) Spatial feature plots of signature score of APOE+CTSZ+TAM and Treg in tissue sections in patients 19 (C) and 36 (D). (E, F) Spearman correlation of signature score of APOE+CTSZ+TAM (*y*‐axis) and Treg (*x*‐axis) in patients 19 (E) and 36 (F).

In order to validate the metabolic and immunosuppressive properties of Macro_APOE/CTSZ in cancer tissues, we used ST data from two CRC patients. In contrast to other spots, *APOE* and *CTSZ* were mainly expressed consistent with *GLUL* expression in the same spot (Fig. 6A,B). Score of Macro_APOE/CTSZ and Treg signatures in each spot highlighted Macro_APOE/CTSZ and Treg may have colocalization in the same spot (Fig. 6C,D). In addition, the signature score of Macro_APOE/CTSZ and Treg showed a significantly positive correlation in two clinical samples (*r* = 0.68, *r* = 0.48, *P* < 2.2e‐16) (Fig. 6E,F).

**Fig. 7:**
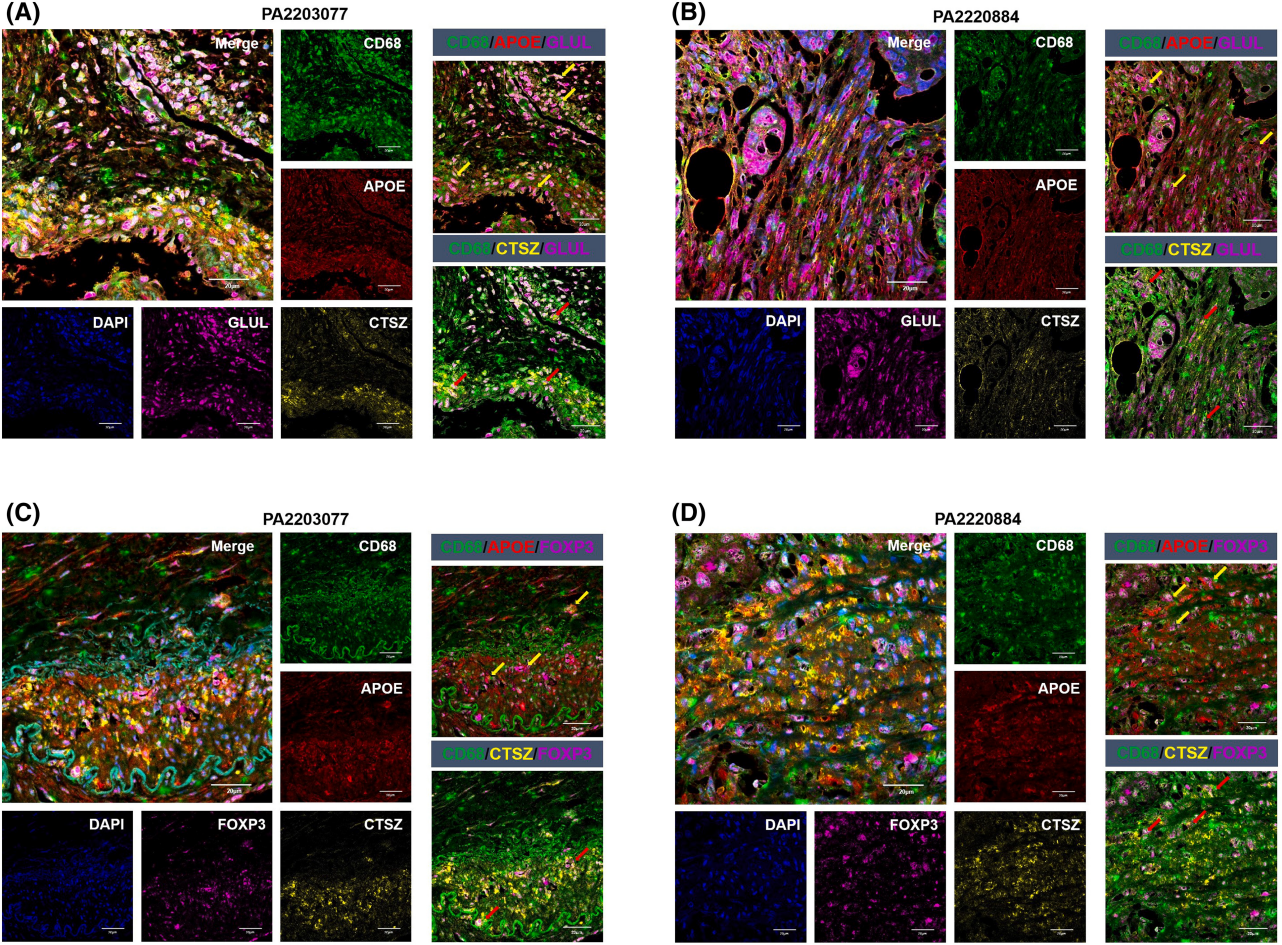
Tissue and cellular distribution of APOE+CTSZ+TAM, GLUL+ cells, and Treg. (A, B) Multiplex immunofluorescence staining of CD68 (green), APOE (red), CTSZ (yellow), GLUL (purple), and DAPI (blue) on CRC tissue section of patient PA2203077 and patient PA2220884, scale bar 20 μm. Left: merged and single‐channel photo of the tissue section. Right: combined channel of CD68/APOE/GLUL and CD68/CTSZ/GLUL on the tissue section. (C, D) Multiplex immunofluorescence staining of CD68 (green), APOE (red), CTSZ (yellow), FOXP3 (purple), and DAPI (blue) on CRC tissue section of patient PA2203077 and patient PA2220884, scale bar 20 μm. Left: Merged and single‐channel photo of the tissue section. Right: Combined channel of CD68/APOE/FOXP3 and CD68/CTSZ/FOXP3 on the tissue section. Arrows indicate the representative regions with three immunofluorescence staining.

To query whether the expression of CD68, APOE, and CTSZ was colocalized with GLUL expression and examine potential spatial interactions between Macro_APOE/CTSZ and Treg within the TME, we performed mIF on available clinical CRC tissue. Immunofluorescent labeling demonstrated CD68/APOE‐positive and CD68/CTSZ‐positive cells localized and overlapped with GLUL‐positive cells (Fig. 7A,B; Fig. S10) and colocalized with FOXP3‐positive cells in nine CRC tissues sections (Fig. 7C,D; Fig. S11). Fluorescence colocalization analysis showed that both Pearson's correlation coefficiency and overlap coefficiency were over 0.80 between APOE/CTSZ and GLUL, and over 0.70 between APOE/CTSZ and FOXP3 (Fig. S10h,i, Table S6), further supporting the potential crosstalk between these two dysfunctional cell populations to promote immunosuppression in malignant CRC.

## 4 Discussion

Through the analysis of myeloid cells, we observed a macrophage subpopulation with the anti‐inflammatory and M2‐like phenotype, which is mainly infiltrated in tumor regions. In addition to the classical anti‐inflammatory markers (such as *APOE*, *CD163*, *CCL18*, and *MSR1*), Macro_APOE/CTSZ is also revealed to express high levels of multiple cysteine cathepsin genes (such as *CTSZ*, *CTSA*, *CTSB*, *CTSD*, and *CTSL*), and complement‐related genes (*C1QC* and *C1QA*). Cysteine cathepsins have been revealed to be associated with extracellular matrix remodeling, antitumor immune response, and protumor behaviors in the previous study [31]. In the current results, Macro_APOE/CTSZ is revealed to constitute the major source of cysteine cathepsin expression in cancer, which may be important for the functional differentiation and immunosuppressive properties of TAM [32]. However, the specific mechanism of cysteine cathepsin needs to be better considered in further studies.

To define the cell states of macrophages clearly, we adopt the marker gene sets related to macrophage function. It is found that Macro_APOE/CTSZ has the highest score of anti‐inflammatory and M2‐like gene signature, which may create the immunosuppressive properties of this cell subtype. Studies have shown that MMRd tumors have high mutation load and usually contain high cytotoxic T‐cell infiltration, while MMRp tumors have low mutation load and less tumor‐associated antigen thus preventing the immune system from activating or inducing less cytotoxic T‐cell infiltration [17]. Combined with the results of the current analysis that Macro_APOE/CTSZ interacts with Treg and is mutually exclusive with T effector cells in CRC‐MMRp, which may indicate that immunosuppressive microenvironment induced by interaction Macro_APOE/CTSZ and Treg may be linked to a low mutation load and deactivating immune system in CRC‐MMRp but not in CRC‐MMRd samples. We then computationally inferred cell–cell communication between above co‐occurred cell types in CRC‐MMRp. The result showed the multiple interactions between Macro_APOE/CTSZ and Treg, including CD74–MIF, CCL3–CCR5, and CXCL16–CXCR6. The chemokine receptor CCR5 has been implicated in the recruitment of Treg from blood into CRC in previous studies [33]. In the current study, *CXCL16* was highly expressed in Macro_APOE/CTSZ compared with other myeloid cells. Previous studies showed that higher *CXCL16* expression was associated with M2‐macrophage infiltration and enhanced angiogenesis in thyroid cancer [34], and TAM promoted cancer metastasis by enhancing CXCL16–CXCR6 pathway in ovarian carcinoma [35]. However, CXCL16–CXCR6 signal has not been studied extensively between TAM and Treg, more exploration needs to reveal the specific mechanism. CD74‐MIF signaling plays an important role in immunosuppression [36], indicating that Macro_APOE/CTSZ may recruit Treg to infiltrate tumor tissue to inhibit antitumor immune responses. ST data and mIF experiment support the inferred interactions between Macro_APOE/CTSZ and Treg in independent cohorts.

Immunosuppressive properties of macrophage are not only controlled at the transcriptional and posttranscriptional level but also influenced by the characteristic of TME, including hypoxia [37] and nutrient availability [38]. As metabolic reprogramming is a hallmark cancer that is characterized by tumor cells altering the metabolic composition of the TME, the recruitment and activation of macrophages can be mediated by functionally metabolic plasticity. Glutamate‐to‐glutamine metabolic pathway and *GLUL* expression, specifically activated and overexpressed in Macro_APOE/CTSZ, were higher in high‐grade compared with low‐grade tumor samples. Glutamine is one of the important and abundant amino acids, which can be used as energy to enter the tricarboxylic acid cycle through production of α‐ketoglutarate [39]. However, how glutamine content impacts the TME are far from being elucidated. It also remains unclear if the underlying mechanisms regulated by glutamate and glutamine metabolism in the TME to mobilize immunosuppressive macrophage function at the single‐cell resolution. Proliferating cancer cells utilize glutamine as an energy‐generating substrate and macrophages also need glutamine metabolism to provide synergistic support for cell activation [40, 41]. Previous studies have identified that GLUL as an enzyme plays a fundamental role in obtaining the prometastatic function of TAM [42] and there is a crosstalk mechanism whereby cancer cells released *N*‐acetylaspartate to enhance GLUL expression in TAM and further prompt M2‐like phenotypes of TAM [43]. Besides, the M2‐like phenotype TAM could be rewired to antitumor and further reduced cancer cell metastasis on mice‐bearing metastatic lung, skin, and breast cancer by GLUL inhibitor [44]. As it is revealed that *SLC1A3* and *GLUL* are highly expressed in Macro_APOE/CTSZ while *SLC1A5* and *SLC38A1* are highly expressed in tumor cells in the current analysis, it may explain that excessive uptake of glutamine by tumor cells creates a shortage of glutamine in the TME, thereby triggering the acquisition of glutamate and synthesis of glutamine by TAM and further facilitating the immunosuppressive phenotype of TAM subpopulation. Combining the previous studies and current analysis, regulating GLUL on macrophages for changing their phenotype and further blocking interaction with Treg to alleviate the immunosuppressive microenvironment, maybe a potential combined treatment strategy with immune checkpoint treatment for CRC and LC patients with high Macro_APOE/CTSZ cell infiltration and who do not respond to immunotherapy, such as MMRp samples [17].

The main limitation of the current study is that additional experimental efforts are needed to establish crosstalk between Macro_APOE/CTSZ and Treg, as well as the connection between Macro_APOE/CTSZ function and glutamate‐to‐glutamine pathway. To determine whether regulating metabolism pathway of glutamate to glutamine in Macro_APOE/CTSZ would effectively abolish the immunosuppressive microenvironment and rehabilitate an antitumor immune response, further research is needed.

## 5 Conclusion

Overall, this study provides probabilities to explore the connection between cellular metabolic heterogeneity and their cellular functions in the TME at single‐cell resolution. The results indicate that Macro_APOE/CTSZ plays a substantial role in the immune dysfunction in higher‐grade tumor, and also assume a metabolic checkpoint of Macro_APOE/CTSZ, which may allow cells to return to antitumor phenotype by targeting pathway of glutamate to glutamine. Ultimately, the interactions we identified between the Macro_APOE/CTSZ and Treg represent potential therapeutic targets, with the goal of further improving antitumor immunity in advanced CRC.

## Conflict of interest

The authors declare no conflict of interest.

## Author contributions

JW designed and conceived the study. JW and WY collected the data and led the data analysis. WY analyzed the single‐cell data. GH analyzed the spatial transcriptome data. JC and LZ performed the multiplex immunofluorescence. JW interpreted the results and wrote the manuscript. ZC evaluated the method. MH and XG contributed to preliminary background research survey and prepared supplemental files. HD conceived of the project, supervised it, and revised the manuscript. All authors read and approved the final version of the manuscript.

### Peer review

The peer review history for this article is available at https://publons.com/publon/10.1002/1878‐0261.13373.

## Supporting information


**Fig. S1.** The cell landscape in the hypoxia tumor microenvironment.
**Fig. S2.** Cell function across cell subtypes.
**Fig. S3.** The metabolic characteristics in single cells.
**Fig. S4.** The metabolic characteristics by pseudo‐bulk analyses in CRC.
**Fig. S5.** The correlation between metabolism and cell function across cell types.
**Fig. S6.** The immunosuppressive function of APOE+CTSZ+TAM.
**Fig. S7.** The glutamate‐to‐glutamine metabolic pathway in APOE+CTSZ+TAM.
**Fig. S8.** Expression pattern of genes involved in glutamate and glutamine metabolic pathway.
**Fig. S9.** The relevance between glutamate‐to‐glutamine metabolic flux and cell function in macrophages.
**Fig. S10.** Tissue and cellular distribution of APOE+CTSZ+TAM and GLUL+cells.
**Fig. S11.** Tissue and cellular distribution of APOE+CTSZ+TAM and Treg.

**Table S1.** Clinical information for patient cohort.

**Table S2.** Markers of cell type and subtypes.

**Table S3.** The cell types and cell numbers.

**Table S4.** Gene set list for ssGSEA analysis.

**Table S5.** Cox regression results for GLUL.

**Table S6.** The results of multiple immunofluorescence quantitative colocalization analysis.

## Data Availability

All data used in this study are publicly available as described in the Materials and methods section. The main source codes for the analysis and visualization of this study are available at the GitHub repository: https://github.com/Dulab2020/singlecell‐analysis.
