# Supplementary material for: Single‐cell and spatial analyses reveal the association between gene expression of glutamine synthetase with the immunosuppressive phenotype of APOE+CTSZ+TAM in cancers
Source: Mol Oncol. 2023 Jan 21;17(4):611–28. doi: 10.1002/1878-0261.13373 (PMC10061288; doi:10.1002/1878-0261.13373)
Supplement: Supplementary file 1 — Fig. S1. The cell landscape in the hypoxia tumor microenvironment. Fig. S2. Cell function across cell subtypes. Fig. S3. The metabolic characteristics in single cells. Fig. S4. The metabolic characteristics by pseudo‐bulk analyses in CRC. Fig. S5. The correlation between metabolism and cell function across cell types. Fig. S6. The immunosuppressive function of APOE+CTSZ+TAM. Fig. S7. The glutamate‐to‐glutamine metabolic pathway in APOE+CTSZ+TAM. Fig. S8. Expression pattern of genes involved in glutamate and glutamine metabolic pathway. Fig. S9. The relevance between glutamate‐to‐glutamine metabolic flux and cell function in macrophages. Fig. S10. Tissue and cellular distribution of APOE+CTSZ+TAM and GLUL+cells. Fig. S11. Tissue and cellular distribution of APOE+CTSZ+TAM and Treg. [file MOL2-17-611-s002.docx]

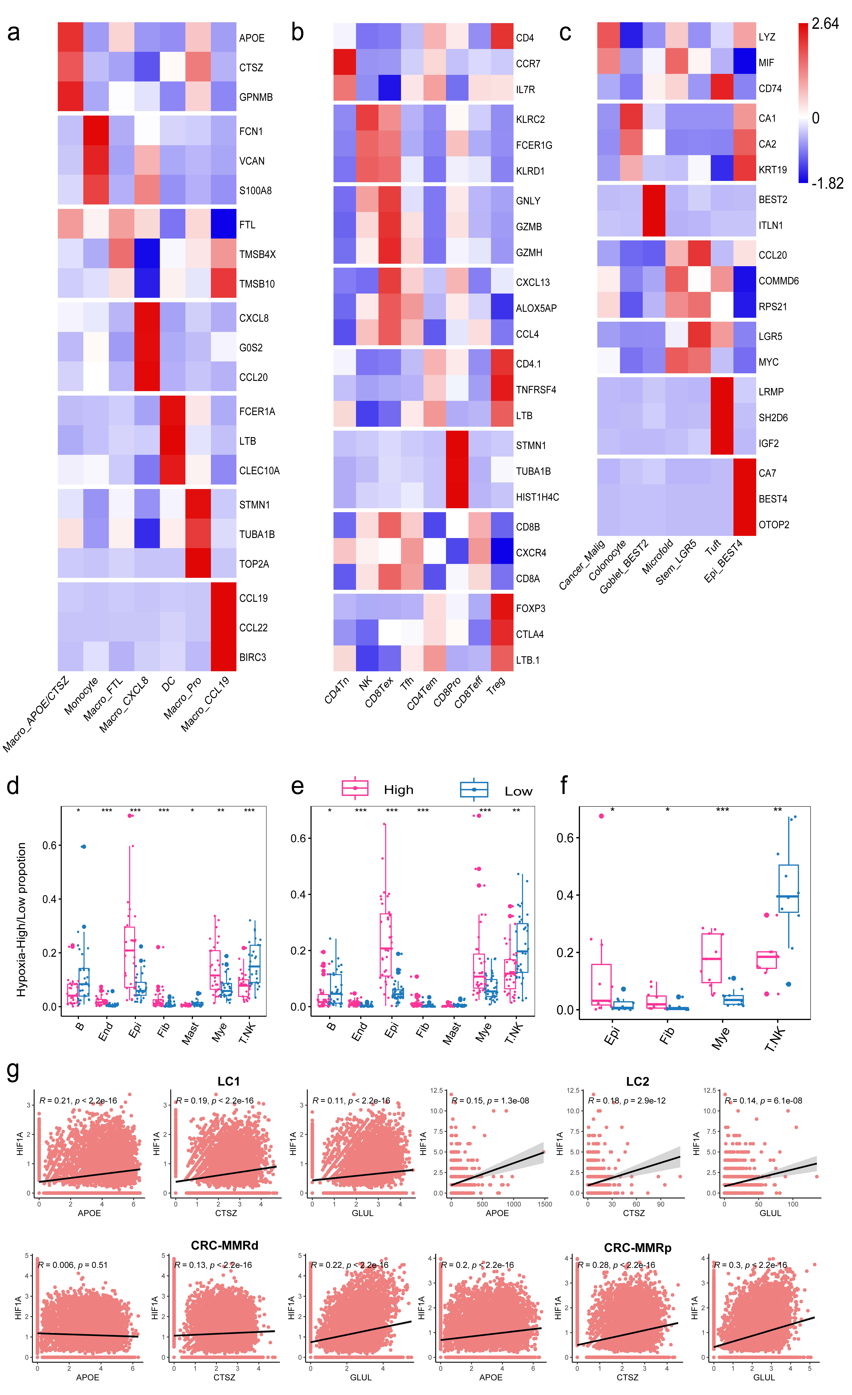


Supplementary Fig 1. The cell landscape in the hypoxia TME. a-c. The subtypes and marker genes of myeloid cell (a), T/NK cells (b) and epithelial cells (c) in CRC-MMRp samples. d-f. Cell proportion in hypoxia high and low groups in CRC-MMRp (d), CRC-MMRd (e) and LC1 (f) samples. Paired two-sided Wilcoxon test, *p < 0.05, **p < 0.01, ***p < 0.01. g. Correlation of gene expression between *HIF1A* with *APOE*, *CTSZ* and *GLUL* in CRC and LC datasets.


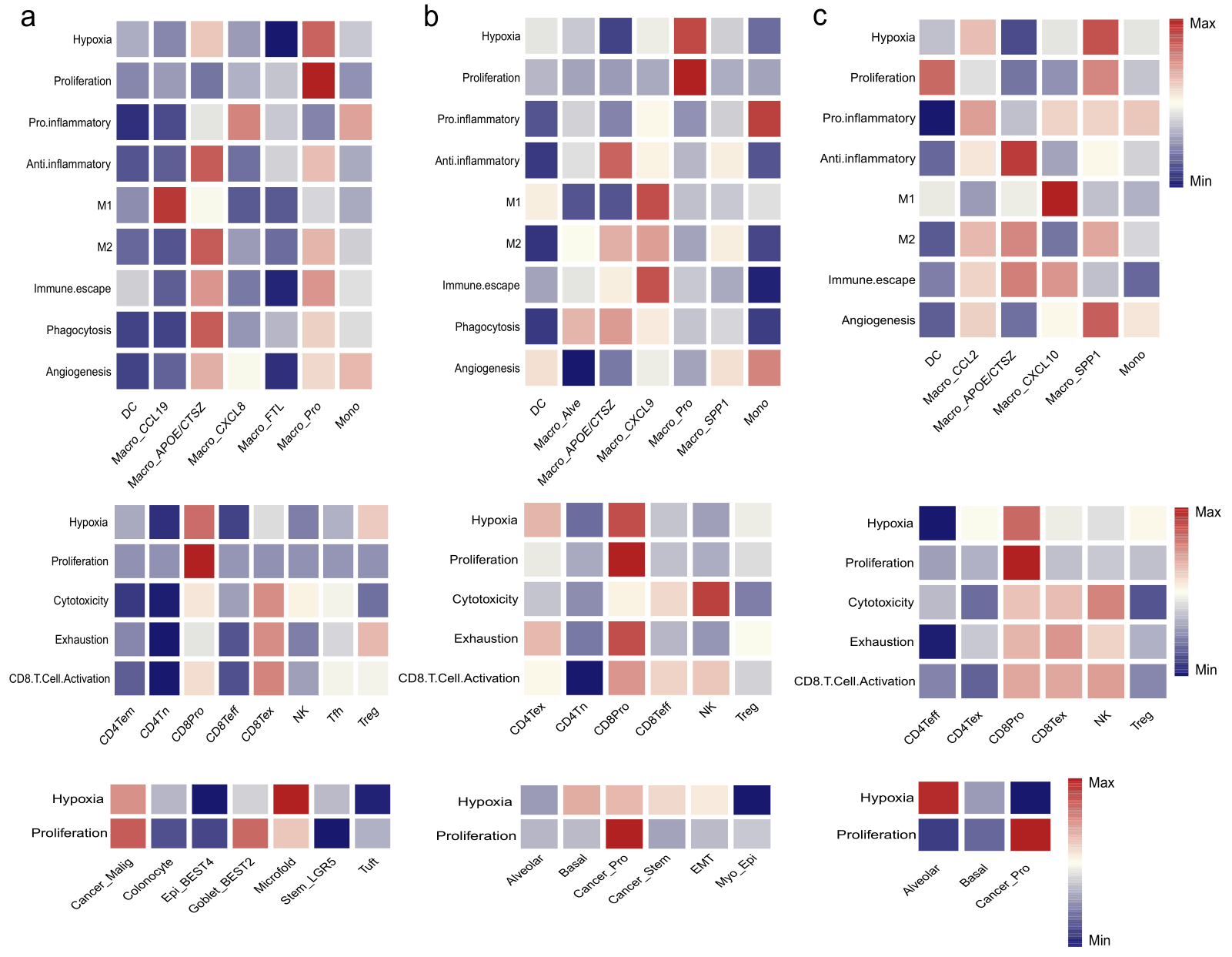


Supplementary Fig 2. Cell function across cell subtypes. a-c. Heatmaps showing different expression patterns of cell function-associated gene signatures among subsets of myeloid cells, T/NK cells and epithelial cells in CRC-MMRd (a), LC1 (b) and LC2 (c) samples.


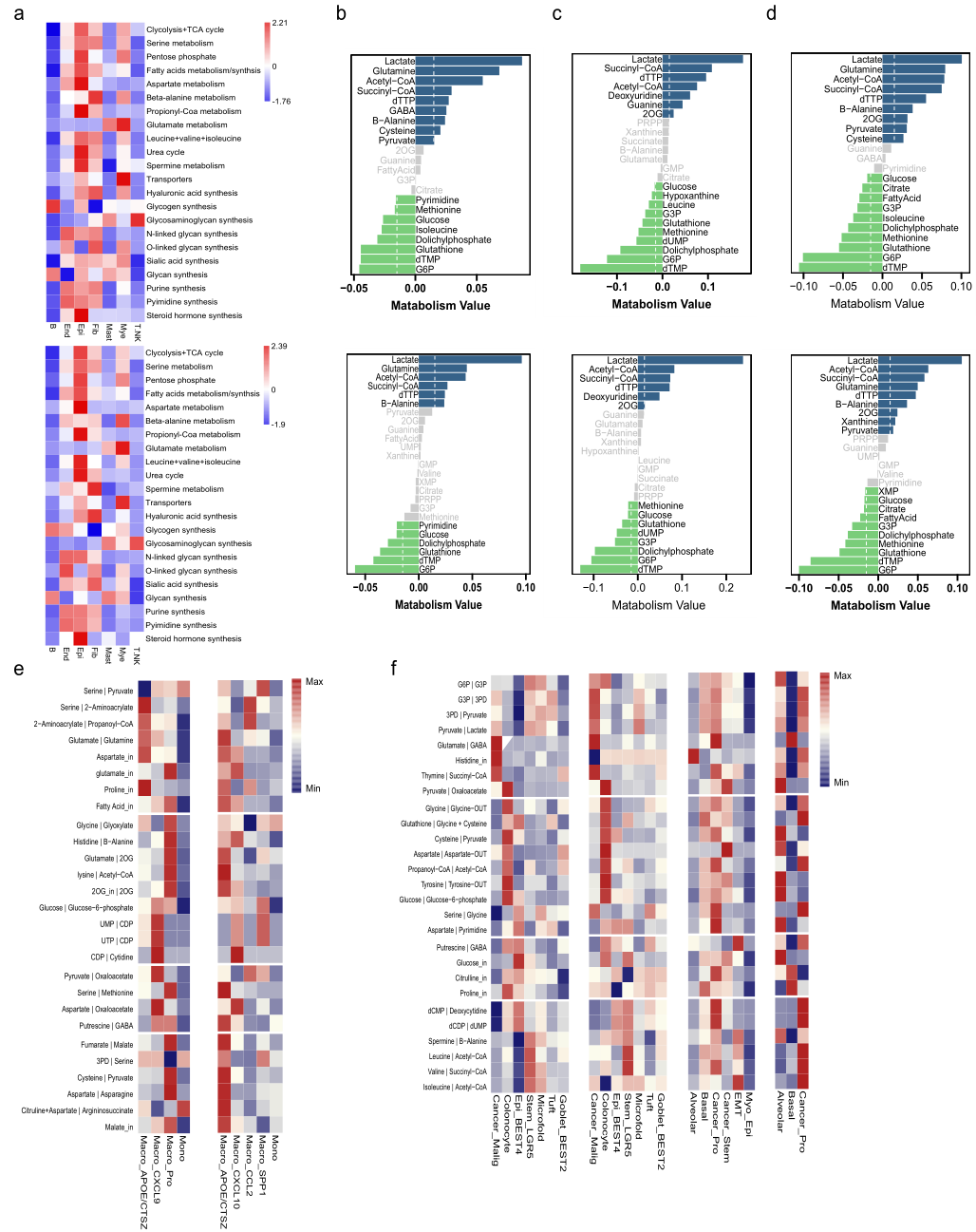


Supplementary Fig 3. The metabolic characteristics in single cells. a. Heatmap showing GSVA score of metabolism pathway between major cell types in CRC-MMRp samples and CRC-MMRd samples. b-d. Top accumulated and depleted metabolites predicted in the myeloid cells (b), epithelial cells (c) and APOE+CTSZ+TAM (d) in LC1 and LC2 samples. e. Distribution of predicted cell-wise flux of metabolism in the subtypes of myeloid cells in LC1 and LC2 samples. f. Distribution of predicted cell-wise flux of metabolism in the subtypes of epithelial cells in CRC-MMRp, CRC-MMRd, LC1 and LC2 samples.


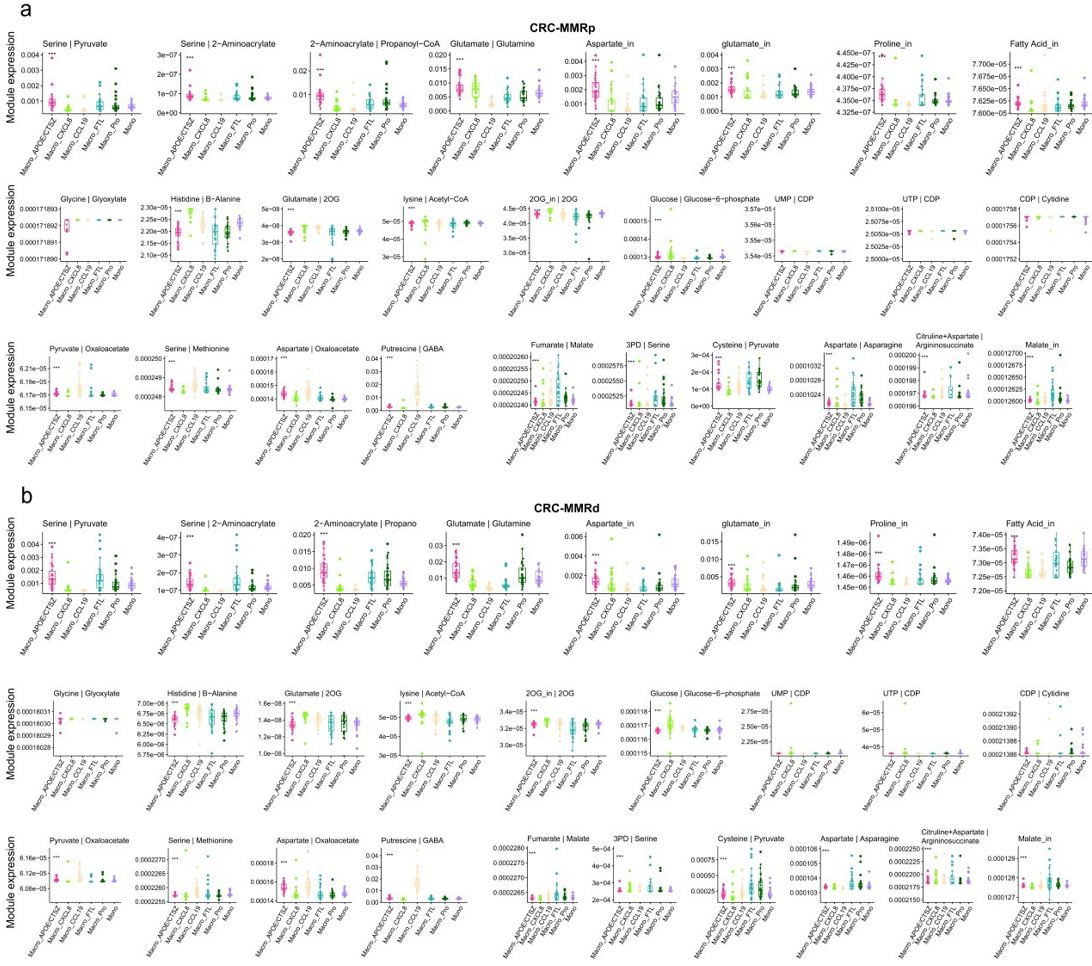


Supplementary Fig 4. The metabolic characteristics by pseudo-bulk analyses in CRC. a, b. Metabolic flux difference between different cell subcluster by grouping cell types from distinct patients for pseudo-bulk analyses in CRC-MMRp samples (a) and CRC-MMRd samples (b).


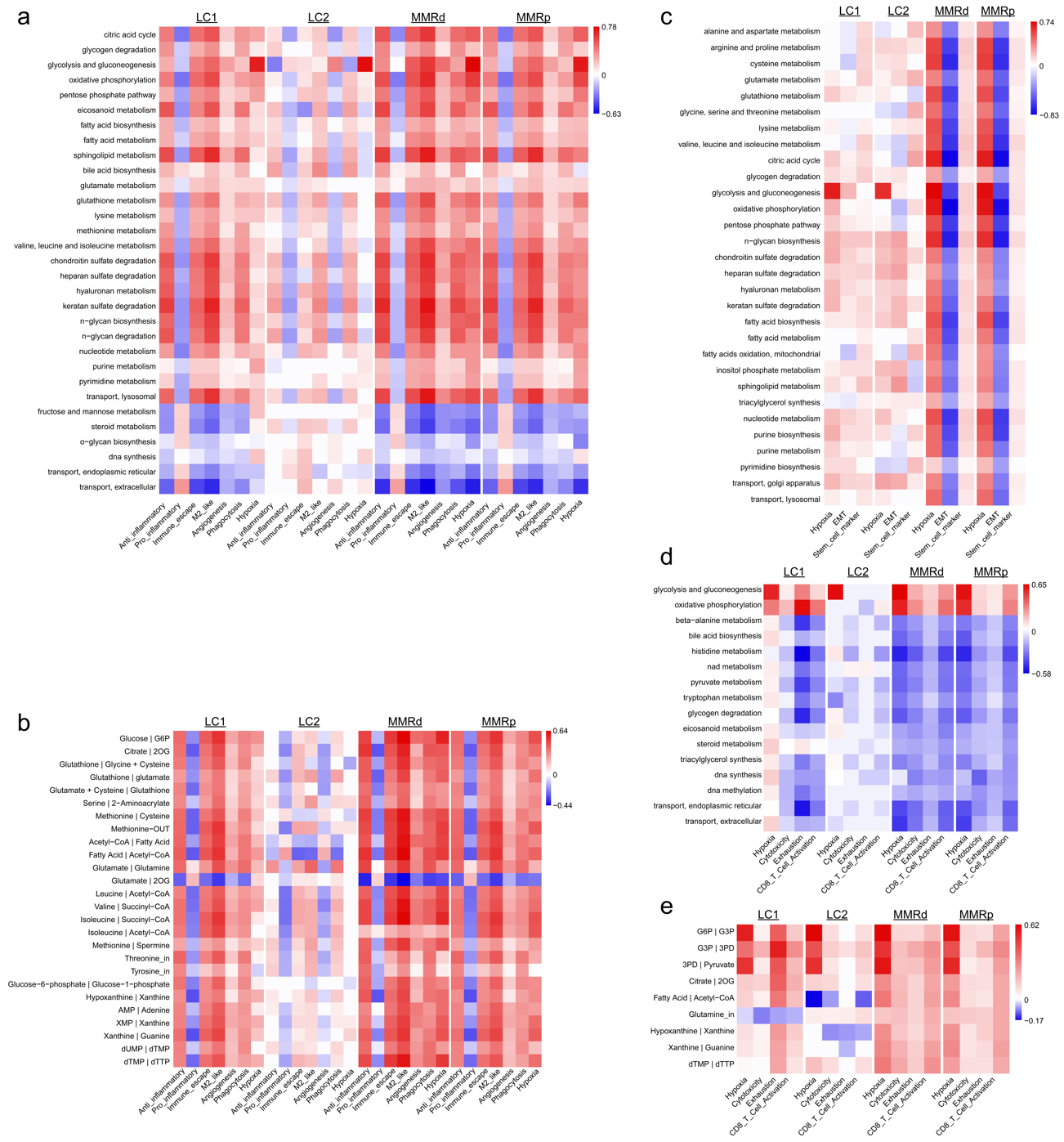


Supplementary Fig 5. The correlation between metabolism and cell function across cell types. a. Heatmaps showing the GSVA score correlation between metabolism pathway and cell function gene sets in myeloid cells. b. Heatmaps showing the correlation between score of metabolism module and GSVA score of cell function gene sets in myeloid cells. c, d. Heatmaps showing the GSVA score correlation between metabolism pathway and cell function gene sets in epithelial cells (c) and T/NK cells (d). e. Heatmaps showing the correlation between score of metabolism module and GSVA score of cell function gene sets in T/NK cells.


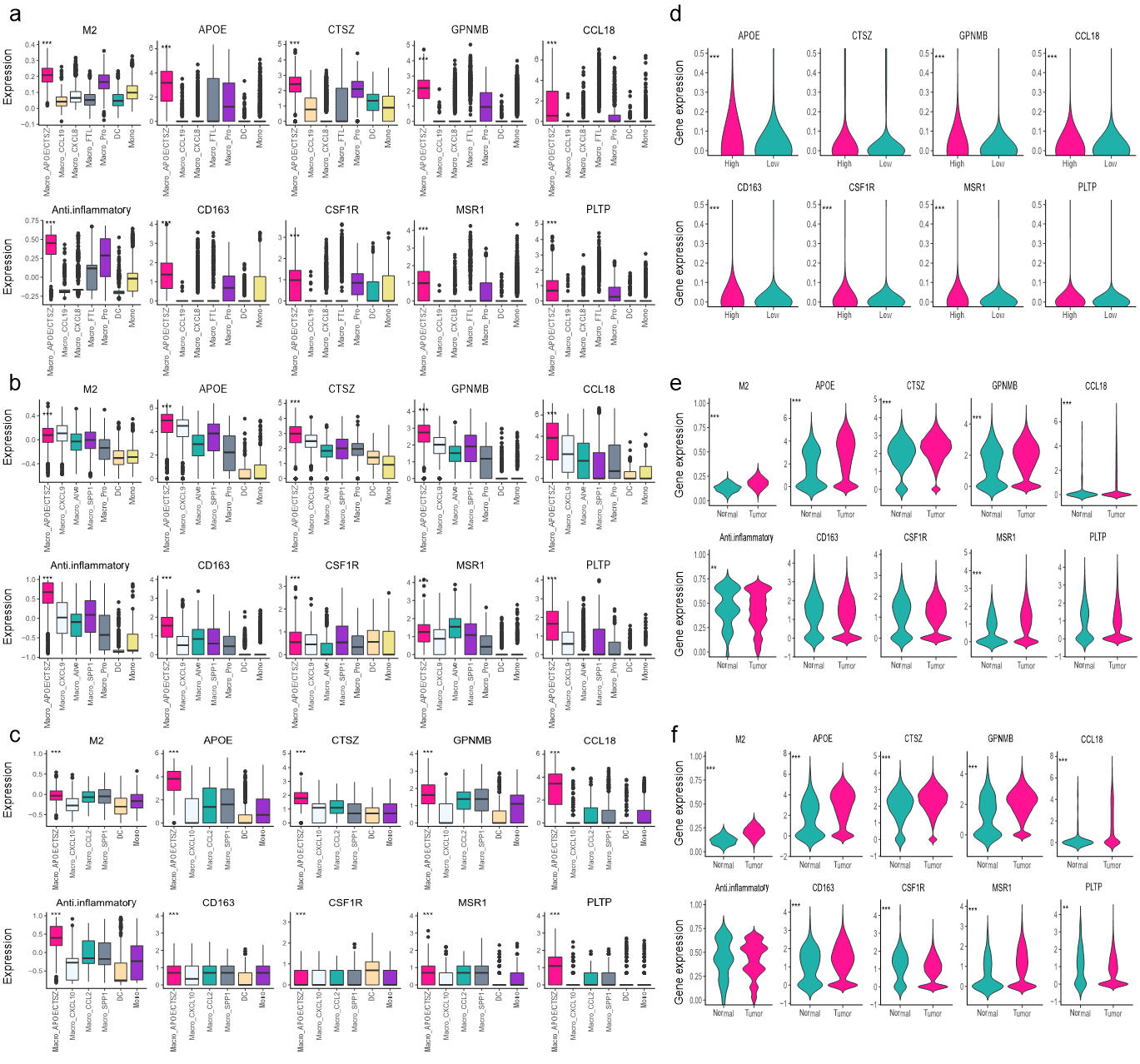


Supplementary Fig 6. The immunosuppressive function of APOE+CTSZ+TAM. a-c. Boxplot showing the different expression of marker genes of APOE+CTSZ+TAM and M2 score as well as anti-inflammatory score among myeloid cells in CRC-MMRd, LC1 and LC2 samples. *** P<0.001, Kruskal-Wallis test. d. Violin plots showing the different expression of marker genes of APOE+CTSZ+TAM between different histologic grade samples in CRC-MMRd samples. *** P<0.001, Paired two-sided Wilcoxon test. e, f. Boxplot showing the different expression of marker genes of APOE+CTSZ+TAM and M2 as well as anti-inflammatory score in myeloid cells derived from normal and tumor samples in CRC-MMRp (e) and in CRC-MMRd (f). ** P<0.01, *** P<0.001, Paired two-sided Wilcoxon test.


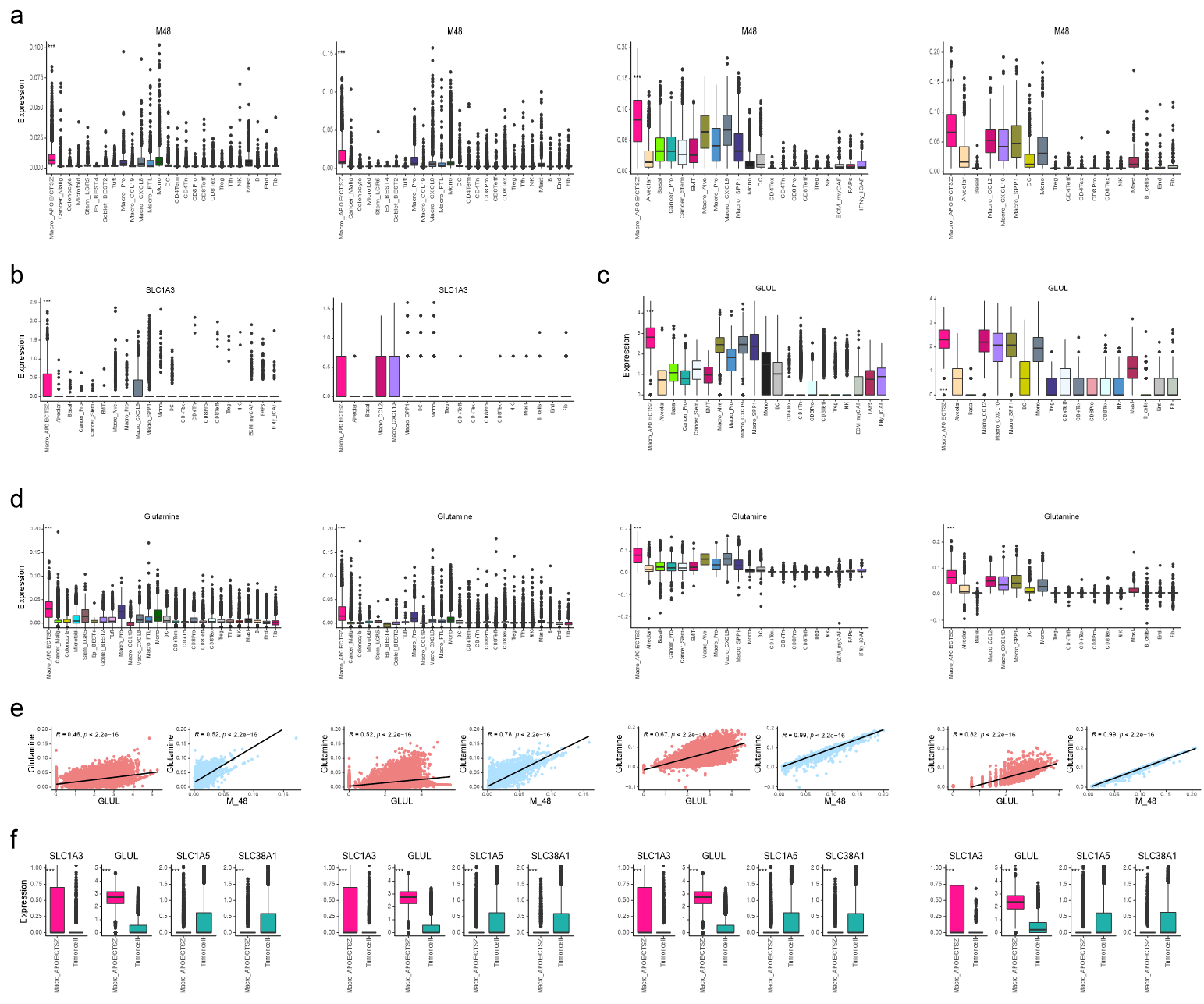


Supplementary Fig 7. The glutamate to glutamine metabolic pathway is highly in APOE+CTSZ+TAMs. a. M_48 score among cell subtypes in CRC-MMRp, CRC-MMRd, LC1 and LC2 samples. b, c. *SLC1A3* (b) and *GLUL* (c) expression among cell subtypes in LC1 and LC2 samples. d. Glutamine accumulation among cell subtypes in CRC-MMRp, CRC-MMRd, LC1 and LC2 samples. e. The correlation between glutamine accumulation and GLUL as well as M_48 in CRC-MMRp, CRC-MMRd, LC1 and LC2 samples. *** P<0.001, Kruskal-Wallis test. f. Boxplot showing the different expression of *SLC1A3*, *GLUL*, *SLC1A5* and *SLC38A1* between APOE+CTSZ+TAM and tumor cells in CRC-MMRp, CRC-MMRd, LC1 and LC2 samples. *** P<0.001, Paired two-sided Wilcoxon test.


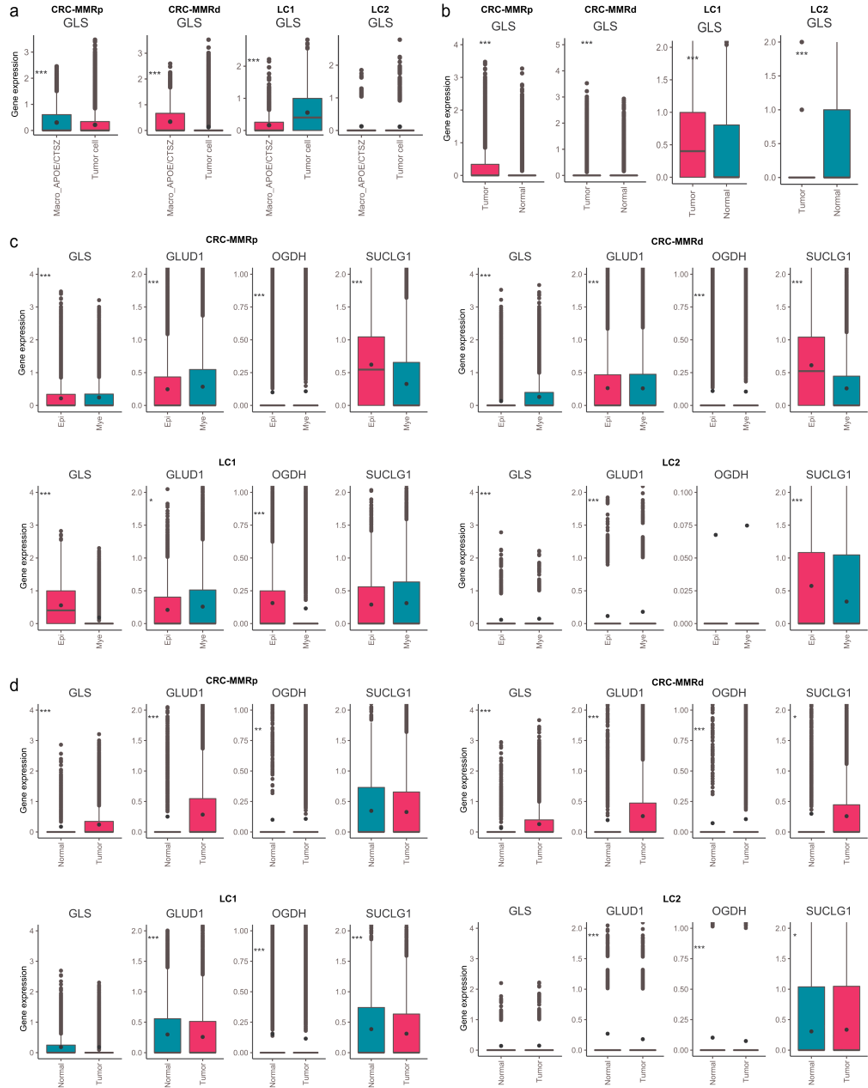


Supplementary Fig.8. Expression pattern of genes involving in glutamate and glutamine metabolic pathway. a. Boxplot showing the different expression of GLS between APOE+CTSZ+TAM with tumor cells in CRC-MMRp, CRC-MMRd, LC1 and LC2 samples. b. Boxplot showing the different expression of GLS between tumor cells derived from tumor with epithelial cells derived from normal samples in CRC-MMRp, CRC-MMRd, LC1 and LC2. c. Boxplot showing the different expression of GLS, GLUD1, OGDH and SUCLG1 between myeloid cells with tumor cells in CRC-MMRp, CRC-MMRd, LC1 and LC2 samples. d. Boxplot showing the different expression of GLS, GLUD1, OGDH and SUCLG1 between myeloid cells derived from tumor with myeloid cells derived from normal samples.


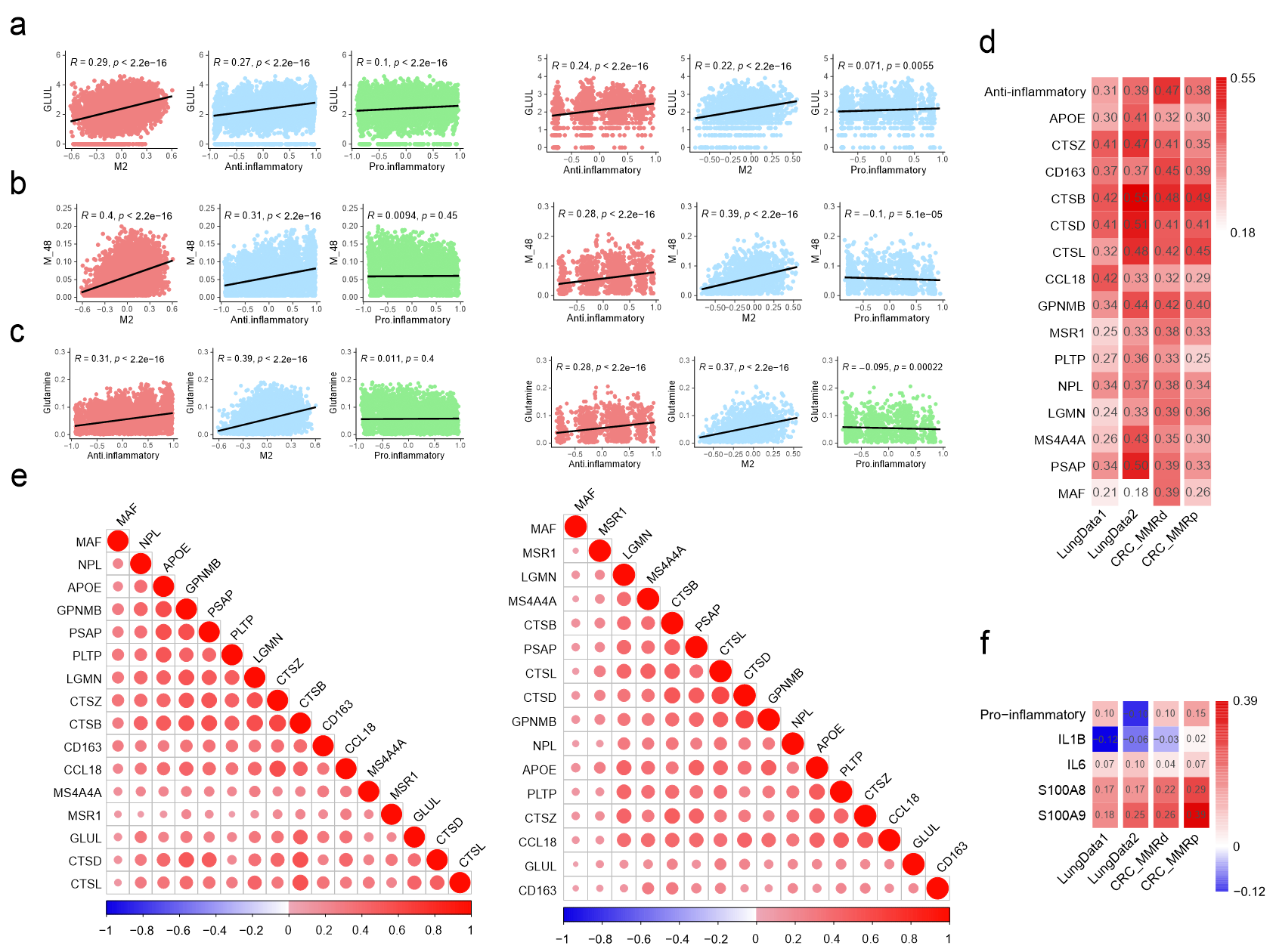


Supplementary Fig 9. The relevance between glutamate to glutamine metabolic flux and cell function in macrophages. a-c. The correlation between *GLUL* expression (a), M_48 score (b) and glutamine accumulation value (c) with anti-inflammatory, M2 and Pro-inflammatory score in LC1 (left) and LC2 (right) samples. d. The correlation between M_48 with genes in anti-inflammatory in LC and CRC samples. e. The correlation between GLUL with genes in anti-inflammatory in LC1 (left) samples and LC2 (right) samples. f. The correlation between GLUL with genes in pro-inflammatory in LC and CRC samples.


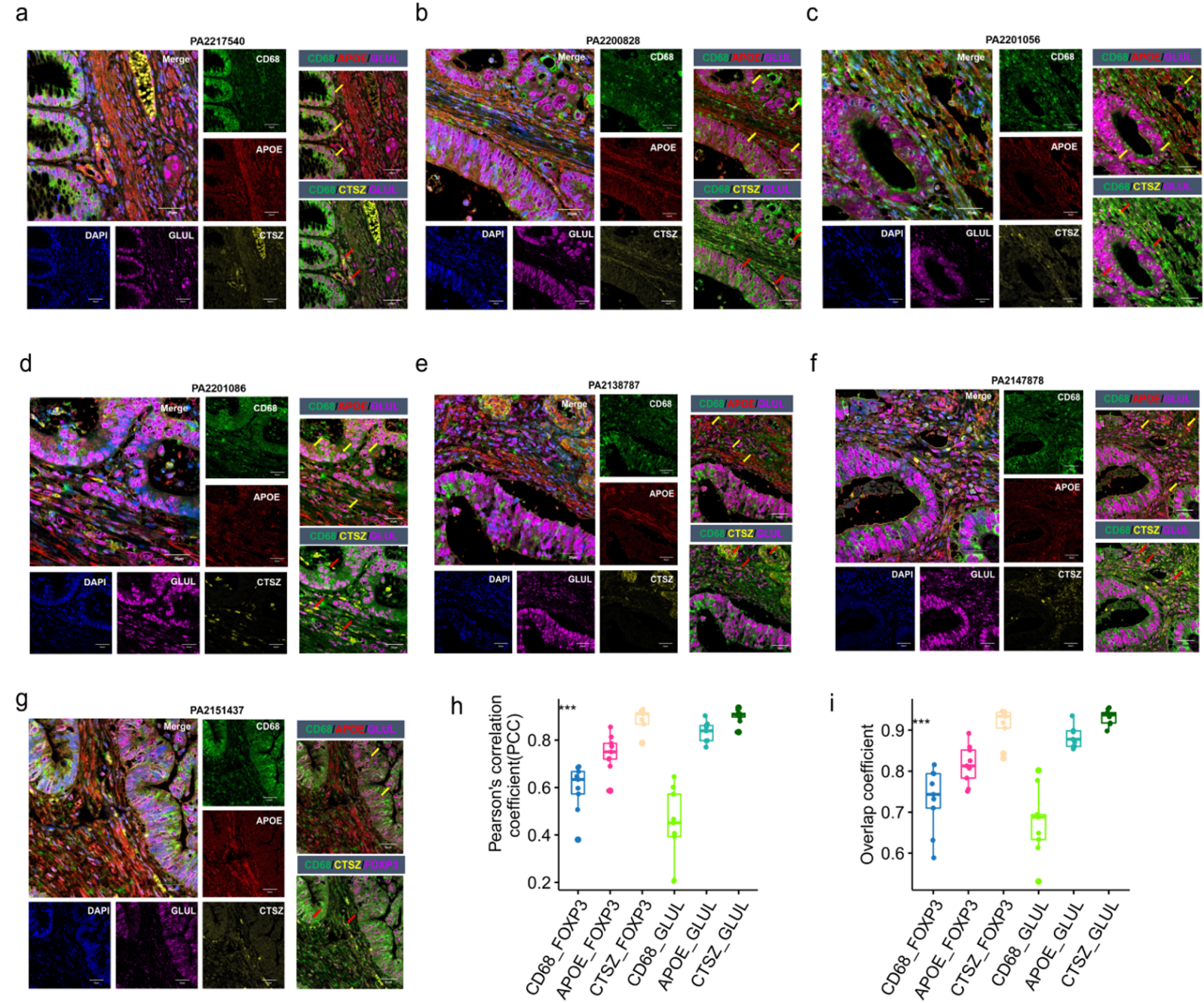


Supplementary Fig 10. Tissue and cellular distribution of APOE+CTSZ+TAM and GLUL+cells. a-g. Multiplex immunofluorescence staining of CD68 (green), APOE (red), CTSZ (yellow), GLUL (purple), and DAPI (blue) on CRC tissue section of patients, scale bar 20 um. Left: merged and single-channel photo of the tissue section. Right: combined channel of CD68/APOE/GLUL and CD68/CTSZ/GLUL on the tissue section. h. Pearson’s correlation coefficiency between markers by immunofluorescence co localization analysis. i. Overlap coefficiency between markers by immunofluorescence co localization analysis. DAPI, diamidino-2- phenylindole dihydrochloride.


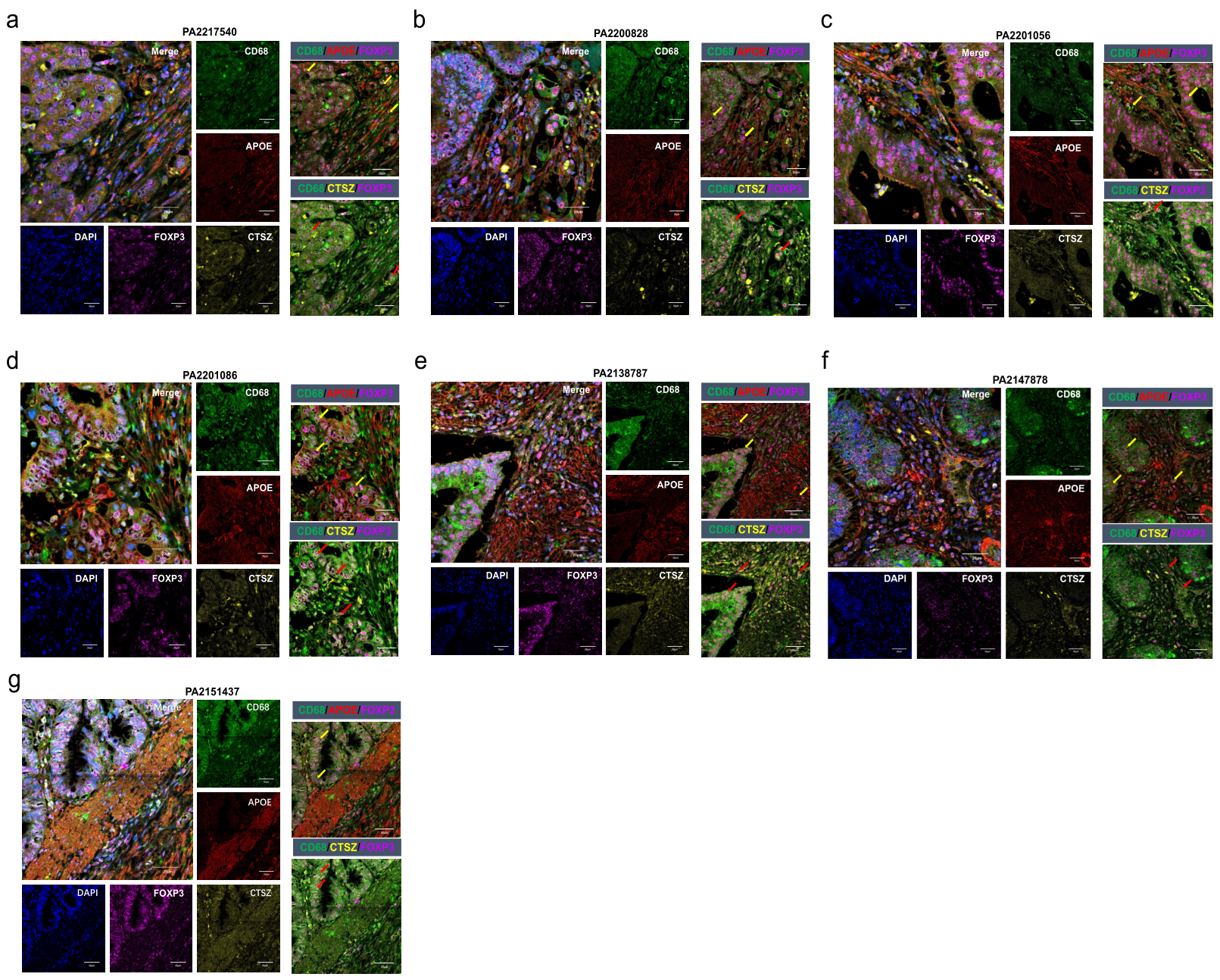


Supplementary Fig 11. Tissue and cellular distribution of APOE+CTSZ+TAM and Treg. a-g. Multiplex immunofluorescence staining of CD68 (green), APOE (red), CTSZ (yellow), FOXP3 (purple), and DAPI (blue) on CRC tissue section of patients, scale bar 20 um. Left: merged and single-channel photo of the tissue section. Right: combined channel of CD68/APOE/FOXP3 and CD68/CTSZ/FOXP3 on the tissue section. APOE_FOXP3, CTSZ_FOXP3, CD68_FOXP3,
